# Pore formation in lipid membrane I: Continuous reversible trajectory from intact bilayer through hydrophobic defect to transversal pore

**DOI:** 10.1038/s41598-017-12127-7

**Published:** 2017-09-22

**Authors:** Sergey A. Akimov, Pavel E. Volynsky, Timur R. Galimzyanov, Peter I. Kuzmin, Konstantin V. Pavlov, Oleg V. Batishchev

**Affiliations:** 1 A.N. Frumkin Institute of Physical Chemistry and Electrochemistry, Russian Academy of Sciences, 31/4 Leninskiy prospekt, Moscow, 119071 Russia; 2 National University of Science and Technology “MISiS”, 4 Leninskiy prospekt, Moscow, 119049 Russia; 3 Shemyakin-Ovchinnikov Institute of Bioorganic Chemistry, Russian Academy of Sciences, 16/10 Miklukho-Maklaya str., Moscow, 117997 Russia; 4 Federal Research and Clinical Center of Physical-Chemical Medicine, 1a Malaya Pirogovskaya, Moscow, 119435 Russia; 5 Moscow Institute of Physics and Technology, 9 Institutsky lane, 141700 Dolgoprudniy, Russia

## Abstract

Lipid membranes serve as effective barriers allowing cells to maintain internal composition differing from that of extracellular medium. Membrane permeation, both natural and artificial, can take place via appearance of transversal pores. The rearrangements of lipids leading to pore formation in the intact membrane are not yet understood in details. We applied continuum elasticity theory to obtain continuous trajectory of pore formation and closure, and analyzed molecular dynamics trajectories of pre-formed pore reseal. We hypothesized that a transversal pore is preceded by a hydrophobic defect: intermediate structure spanning through the membrane, the side walls of which are partially aligned by lipid tails. This prediction was confirmed by our molecular dynamics simulations. Conversion of the hydrophobic defect into the hydrophilic pore required surmounting some energy barrier. A metastable state was found for the hydrophilic pore at the radius of a few nanometers. The dependence of the energy on radius was approximately quadratic for hydrophobic defect and small hydrophilic pore, while for large radii it depended on the radius linearly. The pore energy related to its perimeter, line tension, thus depends of the pore radius. Calculated values of the line tension for large pores were in quantitative agreement with available experimental data.

## Introduction

Lipid bilayer constitutes a major structural component of plasma membranes^1^. Amphiphilic nature of lipid molecules, which contain both polar and hydrophobic parts, determines low permeability of lipid bilayers for broad range of substances and thus allows the membranes to perform barrier function effectively in the cells. Artificial permeabilization of plasma membranes is used for various medical and bioengineering purposes^2–5^. There are two alternative mechanisms of penetration through the membranes: small individual molecules can cross membranes, presumably through local defects of lipid packaging, or water-filled pores through the entire membrane can be formed, enabling non-specific transfer of various polar substances. Herein we focus on the mechanisms of formation of transverse pores in lipid membranes.

The classical pore formation theory^6^ treats a membrane as an infinitely thin film without internal structure subjected to external lateral tension σ_0_. The energy of a cylindrically symmetric pore with the radius of *r* can be expressed as:

1 $$E(r)=2\pi r\gamma -\pi {r}^{2}{\sigma }_{0},$$

where *γ* is the boundary line tension equal to the work performed to create a unit length of pore boundary. The system energy has a maximum at the critical radius of ${r}^{\ast }=\frac{\gamma }{{\sigma }_{0}}$, defining the energy barrier to pore formation $E({r}^{\ast })=\frac{\pi {\gamma }^{2}}{{\sigma }_{0}}$. The pores with the radius below the critical value are reversible and tend to close, whereas the “supercritical” ones grow unlimitedly, eventually causing membrane breakdown. Lateral tension can be controlled in many experimental setups, whereas line tension is essentially defined by elastic properties of the membrane itself^7–9^. The classical theory of pore formation treats the membrane as having no internal structure, and hence does not address the mechanism of pore formation from an originally intact bilayer. Besides that, it assumes line tension to be constant, disregarding possible dependencies on surface tension and on pore radius.

Different experimental techniques have been employed to controllably obtain pores in lipid membranes. The observed values of line tension of the pore boundary depend on the lipid composition, the method used for forming the membrane, and even the lipid manufacturer^10^. A generic value of about 10 pN is usually assumed for the line tension^11^. In refs^12,13^, pores were postulated to evolve from membrane hydrophobic defects in the form of water-filled hydrophobic cylinders occurring through lateral displacements of lipid molecules. The lipid polar heads then slide into the cylinder, lining its inner surface and thus completing formation of the so-called hydrophilic pore. The surface of the hydrophilic pore was assumed torus-shaped^14^, with only bending deformations of the membrane taken into account. This is equivalent to potentially unlimited amount of energy being tacitly added to the system to maintain the postulated shape. Under these conditions, line tension is a non-monotonous function of the pore radius. However, the continuous trajectory of formation of the pore was not considered in the refs^12–14^, only the dependencies of the energy of hydrophobic cylinder and of the hydrophilic pore upon their radii having been considered. At the radius, at which the two energies become equal, transition from the hydrophobic defect into the hydrophilic pore becomes possible in principle, though it can require an additional energy barrier being traversed. Besides that, toroidal shape of the pore boundary surface postulated in ref.^14^ yields the line tension values notably (by a factor of 2–3) exceeding the experimental values. The issue of overestimation of the line tension is not specific to the model of the toroidal shape of the pore boundary surface. Generally, the elasticity theories used for analysis of deformations are only applicable as long as deformations can be considered small, which is not the case for the membrane deformations at the pore boundary^7^. One of the possible methods of accurate evaluation of the line tension is based on the use of microscopic models. A model of pore boundary structure yielding the line tension values consistent with the experimental results was proposed in the work ref.^15^. However, only pores of large (infinite) radius had been considered there, with no analysis of the mechanism of the pore formation.

Elasticity theory of small deformations can be used to calculate pore energy if the surface of the pore boundary is divided into several segments and a reference surface is defined for each segment so that the deviations from such reference surfaces can be considered small throughout each segment^7,8,15^. The surface shapes and deformations are optimized separately for each segment and then continuously conjugated. As we have previously demonstrated, breaking the pore boundary down to as few as three parts is sufficient in the sense that further breakdown does not cause the calculated pore boundary energy to decrease^8^.

The specifics of the molecular organization of lipid molecules at the pore boundary can be investigated with the aid of computer modeling techniques, including molecular dynamic simulations at different levels of discretization. However, the probability of spontaneous formation of the pore on the timescale of the simulations is so small that to the best of our knowledge it was observed in only one work^16^ for a lipid with short hydrocarbon tails, out of which no stable membrane can be physically formed. Pores in such simulations are usually artificially created in unperturbed membranes^16,17^. Thereafter, depending on the purposes of the investigation, pore closure kinetics can be studied, or the pore can be stabilized in a state characterized by a certain radius (or other coordinate describing the pore formation) by means of application of external lateral tension or artificial potential. The results of simulation of closure of the pre-formed pore can be compared against the results of analysis of pore formation trajectories obtained in the frame of the continuum theory of elasticity.

Herein we are suggesting a mechanism of formation of a transverse pore through a lipid bilayer. A complete trajectory is analyzed, starting from intact bilayer through hydrophobic defect to hydrophilic pore. The obtained trajectory is reversible and continuous in the sense that every state of the system is characterized by a single continuous parameter — pore luminal radius. Molecular dynamics methods were employed to obtain pore closure trajectories for the membranes consisting of 1,2-dioleoyl-sn-glycero-3-phosphocholine (DOPC), 1-palmitoyl-2-oleoyl-sn-glycero-3-phosphocholine (POPC), or 1,2-dimyristoyl-sn-glycero-3-phosphocholine (DMPC). They were compared with the trajectories calculated based on continuum theory of elasticity.

## Materials and Methods

### Continuum theory of elasticity

Herein we treat the membrane as a continuous liquid crystal medium. The state of a monolayer is described by a field of unit vectors **n**, known as directors, characterizing time-averaged orientation of lipid molecules. The vector field is defined on a certain surface inside the monolayer parallel to its external boundary, known as a dividing surface^18^. The shape of the surface is defined by a field of unit vectors **N** normal to it. We consider the following three main deformations: 1) splay, quantitatively characterized by divergence of the director along the dividing surface div(**n**), 2) tilt, characterized by deviation of the director from the normal at the given point of the dividing surface **t** = **n** − **N**, and 3) lateral stretch/compression characterized by change of the dividing surface area *a* relative to its area in the initial, non-deformed state *a*_0_, *α* = (*a* − *a*_0_)/*a*_0_. For the sake of generality, lateral tension σ_0_ applied to the membrane is taken into account, although in the present work we limit ourselves by consideration of the case of zero lateral tension only. The case of non-zero lateral tension is considered in the accompanying paper^19^. We assume the lateral tension to be uniform and unaffected by appearance of a pore in the membrane. The deformations are deemed small, so that the energy terms of higher than second order can be neglected. Thus, the energy of a deformed segment of monolayer can be written as^20^:

2 $$W=\int \{\frac{B}{2}{(\text{div}({\bf{n}})+{J}_{0})}^{2}-\frac{B}{2}{J}_{0}^{2}+\frac{{K}_{t}}{2}{{\bf{t}}}^{2}+\frac{{K}_{A}}{2}{\alpha }^{2}+{\sigma }_{0}\}dS-{\sigma }_{0}{A}_{0},$$

where *B*, *K*_*t*_, and *K*_*A*_ are splay, tilt and lateral stretch/compression moduli, respectively; *J*_0_ is the monolayer spontaneous curvature characterizing the preferred shape (curvature) of the monolayer in the absence of external forces and torques, *A*_0_ is the surface area of the monolayer in the initial non-deformed equilibrium state. The sign “+” in front of *J*_0_ is consistent with the conventional definition of the spontaneous curvature, according to which the spontaneous curvature of lysolipids is positive^21^. Indeed, in the absence of tilt, i.e. when **t** = 0, director coincides with normal, **n** = **N**, and div(**n**) = div(**N**) = −*J*, where *J* is geometrical curvature of monolayer surface. Thus, in this case the splay term in Eq. (2) becomes *B*/2(div(**n**) + *J*_0_)^2^ = *B*/2(−*J* + *J*_0_)^2^ = *B*/2(*J* − *J*_0_)^2^, i.e. coincides with Helfrich expression for bending energy^22^. The integration in Eq. (2) is performed over the dividing surface. In this equation, we assign all deformations and elastic moduli to a specific dividing surface, the so-called neutral surface, defined as the surface where energy contributions from splay and lateral stretch/compression deformations are independent of each other. The neutral surface was experimentally found to be located in the proximity of the interface between the lipid polar heads and hydrocarbon tails, at the distance of about 0.7 nm from the external polar surface of the monolayer^18^. We assume the hydrophobic part of the monolayers locally volumetrically incompressible, i.e., any element of the monolayer maintains constant volume during any deformations. This assumption is justified by large values of volumetric compression moduli of membranes^23^. Within the adopted accuracy of approximation, local incompressibility condition reads^20^:

3 $${h}_{{\rm{c}}}=h-\frac{{h}^{2}}{2}\text{div}({\bf{n}})-h\alpha ,$$

where *h*_*c*_ and *h* are thicknesses of monolayer hydrophobic parts in the current and initial, non-deformed state, respectively.

Deformations at the pore boundary defined as deviations from a single reference state cannot be made small by any choice of the reference surface, wherefore equations (2)–(3) do not hold near the pore boundary. A simple way around it is to divide the membrane into several parts so that deformation of each part can be deemed small and conjugate the deformations at the boundaries between the parts based on continuity of the director and neutral surface. The system energy is then minimized varying coordinates of the boundaries between the parts. As demonstrated in the work ref.^8^, dividing the pore boundary into three parts is sufficient in the sense that addition of new parts does not cause any substantial decrease of energy and the calculated line tension values are in good agreement with the experimental data. For a large (infinite) radius pore, the pore boundary was divided only into two such parts in the work ref.^15^. In the present work we also divide the boundary into two parts, which, in comparison with division into three parts, substantially simplifies the analysis and interpretation of the results at the expense of a possible insignificant (by less than 30%) overestimation of the line tension of the boundary.

**Figure 1:**
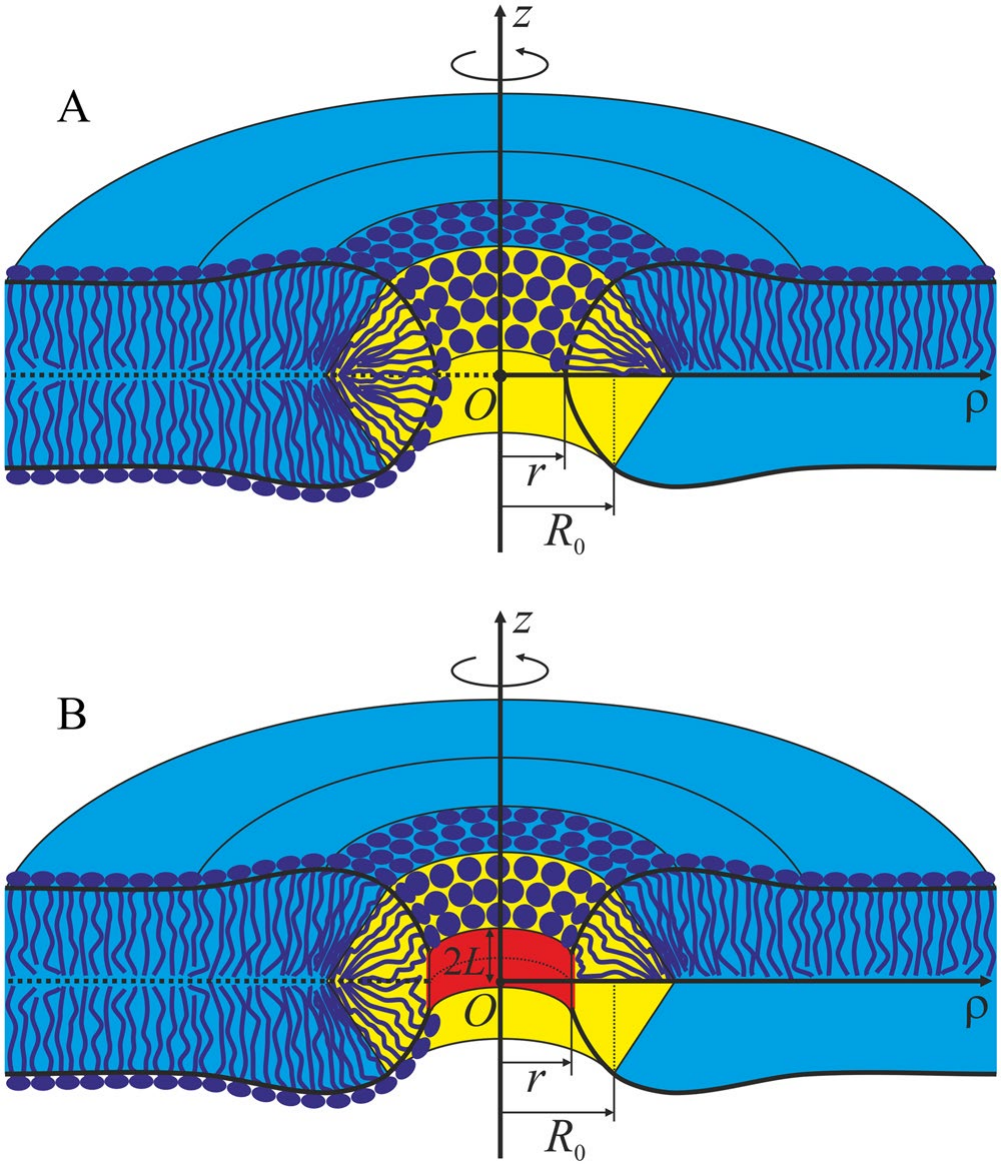
Schematic representation of membrane cross-section by a plane containing the rotational symmetry axis. The horizontal (bilayer) part of the membrane, in which the directors and normals are oriented approximately along the *Oz* axis is shown in blue; the vertical part where the directors and normals weakly deflect from the direction of the *Oρ* axis is highlighted in yellow. The parts are conjugated along two circles of equal radii *R*_0_. The pore radius is designated as *r*. (**A**) hydrophilic pore; (**B**) hydrophobic defect. Cylindrical hydrophobic belt of the height 2*L* and radius *r* is highlighted in red.

For a horizontal membrane with a transverse pore (Fig. 1A), mirror symmetry with respect to the intermonolayer plane and rotational symmetry with respect to a certain axis perpendicular to the membrane surface can be assumed^24,25^. We shall use cylindrical coordinates {*O*, *z*, *ρ*} with the origin *O* in the point of intersection of the rotational symmetry axis with the mirror symmetry plane, *Oz* axis along the rotational symmetry axis and *Oρ* axis perpendicular to it, and divide the pore edge in two parts — a “horizontal bilayer” part where directors and normals weakly deviate from the *Oz* axis direction and a “vertical monolayer” part, where their deviation from the *Oρ* direction is relatively small. The parts are conjugated along a pair of circumferences {*R*_0_, ±*Z*_0_} (Fig. 1A).

Although the division of the pore edge into two parts allows decreasing the artificial overestimation of the calculated elastic energy, it does not completely resolve the limitations of linear theory of elasticity, and the deformations in the vertical monolayer part are still large. As discussed below, at small pore radii large positive meridional curvature is partially compensated by negative equatorial curvature. Nevertheless, application of the linear theory for this edge region cannot be justified *a priory*. However, it is shown experimentally that the linear theory works surprisingly well even for substantial deformations^18,26,27^. In particular, quadratic dependence of splay energy is observed up to the curvatures even higher than the inverse monolayer thickness, keeping splay modulus and position of the neutral surface the same as for small deformations^18^.

#### Horizontal bilayer region

Due to rotational symmetry of the system, all the parameters in this region depend on the *ρ* coordinate only. Therefore, all the vectors can be replaced with their *Oρ* axis projection: **n** → *n*_*ρ*_ = *n*, **N** → *N*_*ρ*_ = *N*, and the director divergence can be, with the adopted accuracy, expressed as div(**n**) ≈ *n*′(*ρ*) + *n*(*ρ*)/*ρ* where the prime in superscript stands for a derivative with respect to *ρ*. Mirror symmetry against the intermonolayer surface allows considering only one monolayer out of the two. The shape of the neutral surface of the upper monolayer can be defined by the distance from the flat intermonolayer surface to the neutral surface as a function *H*(*ρ*). In these notations, the condition (3) of local volumetric incompressibility can be rewritten as follows:

4 $$H=h-\frac{{h}^{2}}{2}(n^{\prime} +\frac{n}{\rho })-h\alpha .$$

Projection of the tilt vector onto the *Oρ* axis equals *t* = *n* − *N* ≈ *n* − *H*′. Expressing the derivative *H*′ from Eq. (4) and substituting director divergence and tilt vector projection into the expression for energy Eq. (2), we obtain the elastic energy functional of the horizontal bilayer region:

5 $$\begin{array}{rcl}{W}_{b} & = & 2{\int }_{{R}_{0}}^{\infty }\pi \rho {K}_{t}\{{l}^{2}{(n^{\prime} +\frac{n}{\rho }+{J}_{0})}^{2}-{l}^{2}{J}_{0}^{2}+{(n+\frac{{h}^{2}}{2}(n^{\prime\prime} +\frac{n^{\prime} }{\rho }-\frac{n}{{\rho }^{2}})+h\alpha ^{\prime} )}^{2}\\  &  & +A{\alpha }^{2}+\sigma {(\frac{{h}^{2}}{2}(n^{\prime\prime} +\frac{n^{\prime} }{\rho }-\frac{n}{{\rho }^{2}})+h\alpha ^{\prime} )}^{2}\}d\rho ,\end{array}$$

where *l* ^2^ = *B*/*K*_*t*_, *A* = *K*_*A*_/*K*_*t*_, σ = σ_0_/*K*_*t*_; the last term under the integral reflects the change of neutral surface area caused by deformations, $(\sqrt{1+{(H^{\prime} )}^{2}}-1)\approx \frac{1}{2}{(H^{\prime} )}^{2}$. The factor of 2 in front of the integral is to take into account both the upper and the lower monolayers in the bilayer region. Variation of this functional with respect to the functions *n*(*ρ*) and *α*(*ρ*) yields the following Euler-Lagrange equations:

6 $$\begin{array}{l}\frac{{h}^{4}}{4}{n}^{(4)}+\frac{{h}^{4}}{2\rho }n\prime\prime\prime +(\frac{{h}^{2}-{l}^{2}}{2\sigma +1}-\frac{3{h}^{4}}{4{\rho }^{2}})n^{\prime\prime} +(\frac{{h}^{2}-{l}^{2}}{2\sigma +1}+\frac{3{h}^{4}}{4{\rho }^{2}})\frac{n^{\prime} }{\rho }\\ \quad \quad \,\,+(\frac{{\rho }^{2}-({h}^{2}-{l}^{2})}{(2\sigma +1){\rho }^{2}}-\frac{3{h}^{4}}{4{\rho }^{4}})n+\frac{{h}^{3}}{2}\alpha \prime\prime\prime +\frac{{h}^{3}}{2\rho }\alpha ^{\prime\prime} +(\frac{h}{2\sigma +1}-\frac{{h}^{3}}{2{\rho }^{2}})\alpha ^{\prime} =0,\\ {h}^{3}n\prime\prime\prime +\frac{2{h}^{3}}{\rho }n^{\prime\prime} +(\frac{2h}{2\sigma +1}-\frac{{h}^{3}}{{\rho }^{2}})n^{\prime} +(\frac{2h}{2\sigma +1}+\frac{{h}^{3}}{{\rho }^{2}})\frac{n}{\rho }\\ \quad \quad \,\,+2{h}^{2}\alpha ^{\prime\prime} +\frac{2{h}^{2}}{\rho }\alpha ^{\prime} -\frac{2A}{2\sigma +1}\alpha =0.\end{array}$$

The general solution of these equations can be written as:

7 $$\begin{array}{rcl}n(\rho ) & = & {g}_{1}{J}_{1}({p}_{1}\rho )+{g}_{2}{J}_{1}({p}_{2}\rho )+{g}_{3}{Y}_{1}({p}_{1}\rho )+{g}_{4}{Y}_{1}({p}_{2}\rho ),\\ \alpha (\rho ) & = & \tfrac{{p}_{1}(2A{l}^{2}-{h}^{2}A+\tfrac{1}{2}{p}_{2}^{2}{h}^{2}({h}^{2}A+4{l}^{2}))}{hA(2+A)}({g}_{1}{J}_{0}({p}_{1}\rho )+{g}_{3}{Y}_{0}({p}_{1}\rho ))\\  &  & +\tfrac{{p}_{2}(2A{l}^{2}-{h}^{2}A+\tfrac{1}{2}{p}_{1}^{2}{h}^{2}({h}^{2}A+4{l}^{2}))}{hA(2+A)}({g}_{2}{J}_{0}({p}_{2}\rho )+{g}_{4}{Y}_{0}({p}_{2}\rho )),\end{array}$$

where *J*_0_, *Y*_0_, *J*_1_, and *Y*_1_ are modified Bessel functions of the order zero and one, respectively; *g*_1_, *g*_2_, *g*_3_, *g*_4_ are complex numbers that have to be determined from the boundary conditions;

8 $${p}_{1,2}=\sqrt{\frac{A({h}^{2}-{l}^{2})-2{h}^{2}\sigma \pm \sqrt{({l}^{4}-2{h}^{4}\sigma -2{h}^{2}{l}^{2}){A}^{2}-4{h}^{2}({l}^{2}+\sigma ({h}^{2}+{l}^{2}))A+4{h}^{4}\sigma }}{{h}^{2}(2\sigma +1)({h}^{2}A+4{l}^{2})}}.$$

The obtained solution (Eq. (7)) can then be substituted into the energy functional Eq. (5). Integration over the neutral surface of the upper monolayer yields the energy of deformed horizontal bilayer region. The resultant expression is analytical, but extremely cumbersome and is therefore not given here.

#### Vertical monolayer region

We characterize the shape of the neutral surface of the vertical monolayer region by the distance from the rotational symmetry axis to the neutral surface *R*(*z*) and by the projection of the unit normal to the surface onto the *Oz* axis, *N*_*z*_(*z*). We use designation *v*(*z*) for director projection onto the *Oz* axis and *β*(*z*) for the relative lateral extension of neutral surface. The distance from the *Oz* axis to the surface lipid tail ends, *M*(*z*), characterizes the shape of this surface. For small deviations of the director from the *Oρ* axis direction div(**n**) ≈ *v*′ + 1/*R*(*z*) where primed character stands for the first derivative with respect to *z*. In these notations, the elastic energy functional reads:

9 $$\begin{array}{rcl}{W}_{m} & = & {\int }_{-{Z}_{0}}^{{Z}_{0}}2\pi R\sqrt{1+{(R^{\prime} )}^{2}}\{\frac{B}{2}{(v^{\prime} +\frac{1}{R}+{J}_{0})}^{2}-\frac{B}{2}{J}_{0}^{2}+\frac{{K}_{t}}{2}{(v-{N}_{z})}^{2}\\  &  & +\frac{{K}_{A}}{2}{\beta }^{2}+{\sigma }_{0}\}dz-2{\sigma }_{0}\pi ({R}_{0}^{2}-{r}^{2}).\end{array}$$

Here *z* arguments are omitted for simplicity; primed symbols stand for derivatives with respect to *z*. The condition of local incompressibility Eq. (3) for the vertical region reads:

10 $$M-R=h-\frac{{h}^{2}}{2}(v^{\prime} +\frac{1}{R})-h\beta .$$

The reference surface for this region is a cylinder coaxial with the *Oz* axis, so that *M*(*z*) and *R*(*z*) can be expressed as: *M*(*z*) = *M*_*v*_ + *m*(*z*); *R*(*z*) = *R*_*v*_ + *u*(*z*) where *M*_*v*_, *R*_*v*_ are positive constants and $|m(z)|$ ≪*M*_*v*_, $|u(z)|$ ≪*R*_*v*_ are small deviations from cylindrical surfaces. In the initial equilibrium the outer, *M*_*v*_, and the inner, *R*_*v*_, radii of the cylinder also have to meet local incompressibility condition:

11 $${M}_{v}-{R}_{v}=h-\frac{{h}^{2}}{2}\frac{1}{{R}_{v}}.$$

Equations (10) and (11) can be combined into an expression for radius deviations, *u*(*z*), to the first order with respect to deformations:

12 $$u(z)=\frac{{R}_{v}^{2}}{2{R}_{v}^{2}+{h}^{2}}(2m(z)+2h\beta (z)+{h}^{2}v^{\prime} (z)).$$

To the same accuracy, the projection of unit normal on the *Oρ* axis *N*_*z*_ = −*u*′(*z*). Substituting this expression along with Eqs (10)–(12) into the energy functional Eq. (9), truncating Taylor series expansion with respect to *v*(*z*), *m*(*z*), β(*z*) functions at the second order and minimizing the obtained functional with respect to these functions, we obtain a system of three Euler-Lagrange differential equations. Since the functional is of the second order in deformations, the equations turn out linear and can be solved analytically. Omitting straightforward but tedious calculus, the general solution of Euler-Lagrange equations can be written as:

13 $$\begin{array}{c}\beta (z)=0,\\ v(z)={d}_{1}{e}^{-{q}_{1}z}+{d}_{2}{e}^{{q}_{1}z}+{d}_{3}{e}^{-{q}_{2}z}+{d}_{4}{e}^{{q}_{2}z},\\ m(z)=\frac{(2{R}_{v}^{2}+{h}^{2})({l}^{2}-2\sigma {R}_{v}^{2})}{4{R}_{v}{l}^{2}}+{D}_{1}{d}_{1}{e}^{-{q}_{1}z}+{D}_{2}{d}_{2}{e}^{{q}_{1}z}+{D}_{3}{d}_{3}{e}^{-{q}_{2}z}+{D}_{4}{d}_{4}{e}^{{q}_{2}z},\end{array}$$

where *d*_1_, *d*_2_, *d*_3_, *d*_4_ are complex coefficients determined from the boundary conditions; *q*_1_, *q*_2_ are complex reciprocal characteristic lengths of change of deformations; *D*_1_, *D*_2_, *D*_3_, *D*_4_ are known constants expressed through different combinations of membrane parameters.

#### Boundary conditions. Conjugation of the regions of the pore boundary

The solutions obtained in the horizontal and vertical regions must be conjugated along the circumferences {*R*_0_, *Z*_0_} and {*R*_0_, −*Z*_0_} delineating them based on the considerations of continuity of neutral surfaces and director. To the first order of deformations, the boundary conditions read:

14 $$H({R}_{0})={Z}_{0},R({Z}_{0})={R}_{0},n({R}_{0})-v({Z}_{0})=1$$

where *n* is *Oρ* projection of the director, *v* — its *Oz* projection. Besides that, all the functions must be real for any real values of *z* and *ρ*. It imposes a set of constraints on real and imaginary parts of *g*_1_, *g*_2_, *g*_3_, *g*_4_ and *d*_1_, *d*_2_, *d*_3_, *d*_4_. The horizontal bilayer region is assumed undeformed at large distances from the pore, yielding the boundary conditions:

15 $$n(\rho =\infty )=0,n\text{'}(\rho =\infty )=0,H(\rho =\infty )=h.$$

Due to mirror symmetry against intermonolayer surface, *v*(*z* = 0) = 0. The pore is parametrized by the radius *r* in the equatorial plane, that is *R*(*z* = 0) = *r*. The remaining indefinite coefficients are found from the condition of minimum of the total energy

16 $$W={W}_{b}+{W}_{m}\mbox{--}\pi {r}^{2}(2{\sigma }_{0}).$$

#### Hydrophobic defect

It is assumed that hydrophilic pore is formed in the originally intact bilayer through an intermediate state referred to as a hydrophobic defect. We postulate it to consist of a horizontal bilayer region, vertical monolayer region, and cylindrical hydrophobic belt of the height of 2*L* and radius *r*, coaxial with *Oz* (Fig. 1B). The energy of water-filled hydrophobic cylinder is calculated in the refs^13,28^ based on Marcelja theory^29^. In our notation system the energy reads:

17 $${W}_{h}=(4\pi rL){\sigma }_{h}\frac{{I}_{1}(\frac{r}{{\xi }_{h}})}{{I}_{0}(\frac{r}{{\xi }_{h}})},$$

where (4*πrL*) is the cylinder side surface area; σ_*h*_ is macroscopic lateral tension at the surface separating lipid tails and water; *ξ*_*h*_ ∼ 1 nm is characteristic length of hydrophobic interactions^30^; *I*_0_, *I*_1_ are Bessel functions of order zero and one, respectively. For the vertical region, the boundary condition in the equatorial plane (*v*(*z* = 0) = 0, *R*(*z* = 0) = *r*) is replaced with the condition at the edge of the hydrophobic belt:

18 $$\begin{array}{c}R(L)=r,\\ v(L)=\frac{-L}{\sqrt{{L}^{2}+{(h-L)}^{2}}}.\end{array}$$

The latter condition is a direct consequence of local incompressibility, i.e. constant density of lipid hydrocarbon chains, applied to the hydrophobic belt^26–28^. Elastic energy functional of the vertical monolayer region, Eq. (9), in this case reads:

19 $$\begin{array}{rcl}{W}_{m} & = & 2{\int }_{L}^{{Z}_{0}}2\pi R\sqrt{1+{(R^{\prime} )}^{2}}\{\frac{B}{2}{(v^{\prime} +\frac{1}{R}+{J}_{0})}^{2}-\frac{B}{2}{J}_{0}^{2}+\frac{{K}_{t}}{2}{(v-{N}_{z})}^{2}\\  &  & +\frac{{K}_{A}}{2}{\beta }^{2}+{\sigma }_{0}\}dz-2{\sigma }_{0}\pi ({R}_{0}^{2}-{r}^{2}),\end{array}$$

where the multiplier 2 reflects two symmetrical monolayer sections above and below the hydrophobic belt. Total energy of the membrane with the hydrophobic defect equals:

20 $$W={W}_{b}+{W}_{m}+{W}_{h}\,-\,\pi {r}^{2}(2{\sigma }_{0}).$$

#### Optimization of deformations at the pore boundary

To find the values of *R*_0_ and *Z*_0_ characterizing the circumferences conjugating the vertical and horizontal regions, we minimize the total free energy of the pore, Eq. (16), or hydrophobic defect, Eq. (20), under variable *R*_0_ and *Z*_0_ using gradient descend method. Certain starting values *R*_0_^0^ and *Z*_0_^0^ are selected, and the free energy gradient of the system is iteratively calculated as:

21 $${\rm{grad}}(W)=[\begin{array}{c}\frac{W({R}_{0}^{[i]}+{\delta }_{0},{Z}_{0}^{[i]})-W({R}_{0}^{[i]},{Z}_{0}^{[i]})}{{\delta }_{0}}\\ \frac{W({R}_{0}^{[i]},{Z}_{0}^{[i]}+{\delta }_{0})-W({R}_{0}^{[i]},{Z}_{0}^{[i]})}{{\delta }_{0}}\end{array}],$$

where *δ*_0_ is a small increment (we used *δ*_0_ = 10^−15^ nm) and *R*_0_^[*i*]^, *Z*_0_^[*i*]^ are *i*-th iterations of *R*_0_ and *Z*_0_. The (*i* + 1)-th iteration of the coordinates is defined as:

22 $$[\begin{array}{c}{R}_{0}^{[i+1]}\\ {Z}_{0}^{[i+1]}\end{array}]=[\begin{array}{c}{R}_{0}^{[i]}\\ {Z}_{0}^{[i]}\end{array}]-{\delta }_{C}{\rm{grad}}(W),$$

where *δ*_*C*_ is a constant selected so as to ensure convergence of the algorithm. The gradient descend continued until |grad(*W*)| became less than 10^−7^ *k*_*B*_ *T*/nm (*k*_*B*_ *T* ∼ 4.14·10^−21^ J). It is worth pointing out that though application of Euler-Lagrange formalism to our model yields analytical expression for pore energy under given boundary conditions, the position of the boundary itself affects the resultant pore energy. Optimization of energy with respect to this parameter {*R*_0_, ±*Z*_0_} was performed numerically using gradient descent method, hence no analytical expression was obtained for the final optimized energy of the pore, and consequently for the line tension.

### Molecular dynamics

Molecular dynamics (MD) simulations were performed with Gromacs 4.6^31^ using Slipids^32^ force field and tip3p water model^33^. At the first stage of analysis, equilibrium properties of bilayers composed of either of the three lipids (DOPC, POPC or DMPC) were modeled. Starting configuration of the system was created by translation of a single lipid molecule to a 10 × 10 grid with the step of 0.8 nm. Then the system was solvated and equilibrated by 1000 steps of steepest descent energy minimization followed by 250 ps MD with constant temperature (310 K, Nose-Hoover thermostat^34^) and semiisotropic pressure (1 bar, Berendsen barostat^35^). The bilayer was then equilibrated through a 100 ns MD run at constant temperature and pressure. Temperature was maintained at 310 K with Nose-Hoover thermostat^34^. Pressure was controlled semi-isotropically with Parrinello-Rahman barostat^36^. The MD integration step was 0.002 ps; van der Waals interactions were truncated using twin range 1.0/1.4 nm spherical cut-off. Electrostatic effects were treated using particle-mesh Ewald scheme. The second half of the trajectory was used to obtain lateral diffusion coefficient and evaluate the accessible hydrophobic surface part at equilibrium.

The second stage of the analysis included simulation of pore closure. Starting configuration of the system was created by replication of the equilibrated bilayer obtained at the first stage to a 3 × 3 grid with the central cell removed. Such a large system allows avoiding possible artifacts originating from periodic boundary conditions appearing as interaction of pores in the adjacent simulation boxes, and generation of extraneous bending torques at the box boundaries. After solvation of the system, its behavior was studied in 50 ns MD runs with the parameters similar to those used at the first stage. Two independent trajectories were obtained for each lipid. The following parameters were analyzed: the mean pore radius <*r*>, the hydrophobic (*S*_*phob*_) and the hydrophilic accessible (*S*_*phil*_) surface as functions of time with the sampling interval of 0.1 ps. Free volume in the bilayer was analyzed to identify pores. Cavities in the membrane were initially identified from the analysis of 3D-density grid the with cell size of 3.4 Å. Then, the cavities overlapping in the membrane plane were combined together and the largest cavity was analyzed as a pore. A dot Connolly surface of the membrane with the dot density of 3 per Å^2^ was then calculated. The surface points located in the grid cells of pore cavity and adjacent cells were considered to form the pore boundary (set *S*). Then, in order to evaluate radius and hydrophobicity profiles the pore was decomposed into parallel layers *S*_*z*_ [*z* − *dz*; *z* + *dz*]. The pore center was defined as average location of all the dots included in the layer, and the pore radius *R*(*z*) — as the averaged distance from the surface points to the center. Pore hydrophobicity was characterized by the mean value of hydrophobic potential induced by the bilayer atoms as described in the ref.^37^. The profiles were analyzed within 15 Å from the membrane center with the step of 0.5 Å. A similar approach to pore analysis was recently applied for characterization of protein pores^38^. The dependence of pore hydrophobicity on its radius, *S*_*phob*_(*r*), was obtained as a parametric function from the dependencies <*r*(*t*)> and *S*_*phob*_(*t*).

## Results

### System parameters

Herein, we consider the case of zero lateral tension (σ_0_ = 0) only. The effect of the applied lateral tension is analyzed in the accompanying paper^19^. The results obtained with the aid of continuum theory of elasticity are illustrated for a generic model lipid, DOPC, POPC, and DMPC. The elastic parameters of model lipids are designated by the index “*m*”. The reference model lipid is assumed to have the following parameters: splay modulus (per monolayer) *B*_*m*_ = 8 *k*_*B*_ *T*; lateral stretch/compression modulus (per monolayer) *K*_*A*_^*m*^ = 100 mN/m; thickness of the hydrophobic part of the monolayer *h*_*m*_ = 2 nm, spontaneous curvature *J*_0_ = 0. To illustrate the effects of spontaneous curvature, model lipids with *J*_*m*_ = −0.1 nm^−1^ and +0.1 nm^−1^ are considered, other parameters being the same. To analyze the influence of other parameters on the pore edge energy, we consider model lipids with the elastic moduli and monolayer thickness 1.5 times smaller and larger than those of the reference model lipid. Specifically, the values of *h*_*m*_ = 1.3 nm and 3 nm for membrane thickness, *B*_*m*_ = 5.3 *k*_*B*_ *T* and 12 *k*_*B*_ *T* for splay modulus, and *K*_*A*_^*m*^ = 67 mN/m and 150 mN/m for stretch/compression modulus were used, other parameters being the same as those of the reference model lipid.

For DOPC, the following values of elastic parameters are experimentally determined: splay modulus (per monolayer) *B* = 10.3 ± 1.2 *k*_*B*_ *T* ^39^; lateral stretch/compression modulus (per monolayer) *K*_*A*_ = 133 ± 9 mN/m^39^; thickness of the hydrophobic part of the monolayer *h* = 1.45 ± 0.02 nm^39^; spontaneous curvature *J*_*DOPC*_ = −0.091 ± 0.008 нм^−1^ (ref.^40^) or −0.11 nm^−1^ (ref.^18^); we use the value *J*_*DOPC*_ = −0.091 nm^−1^. The parameters measured for POPC are as follows: splay modulus (per monolayer) *B* = 11 *k*_*B*_ *T* ^39,41^; lateral stretch/compression modulus (per monolayer) *K*_*A*_ = 117 mN/m^39^; thickness of the hydrophobic part of the monolayer *h* = 1.46 nm^42^; spontaneous curvature *J*_*POPC*_ = −0.022 nm^−1^ (ref.^40^). The parameters measured for DMPC are as follows: splay modulus (per monolayer) *B* = 6.8 *k*_*B*_ *T* ^39^; lateral stretch/compression modulus (per monolayer) *K*_*A*_ = 117 mN/m^39^; thickness of the hydrophobic part of the monolayer *h* = 1.37 nm^39^. To the best of our knowledge, the DMPC spontaneous curvature has not been reported. However, the measured spontaneous curvature of saturated lipid 1,2-dipalmitoyl-sn-glycero-3-phosphocholine (DPPC) is *J*_*DPPC*_ = +0.068 nm^−1^ (ref.^40^). DMPC has a smaller volume of the hydrophobic part of the molecules, so it should have a somewhat more positive spontaneous curvature. Therefore, we assumed that DMPC spontaneous curvature is equal to *J*_*DMPC*_ = +0.075 nm^−1^. Equal tilt modulus of *K*_*t*_ = 40 mN/m (per monolayer) was assumed for all lipids^20,43^.

In ref.^30^, the characteristic length of hydrophobic interactions *ξ*_*h*_ = 1 nm was measured. However, in these experiments the hydrophobic surface was rather rigidly bound to a solid support, restricting radial displacements possible for the hydrocarbon tails of the lipids forming the hydrophobic belt surface. Such displacements can cause *ξ*_*h*_ to increase. In order to take into account the possibility of such displacements, we made calculations for two values of the characteristic length of hydrophobic interactions: *ξ*_*h*_ = 1 nm and *ξ*_*h*_ = 1.5 nm. The surface tension at the interface between lipid tails and water was assumed at σ_*h*_ = 36 mN/m^44^.

### Molecular dynamics modeling of pore spontaneous closure

**Figure 2:**
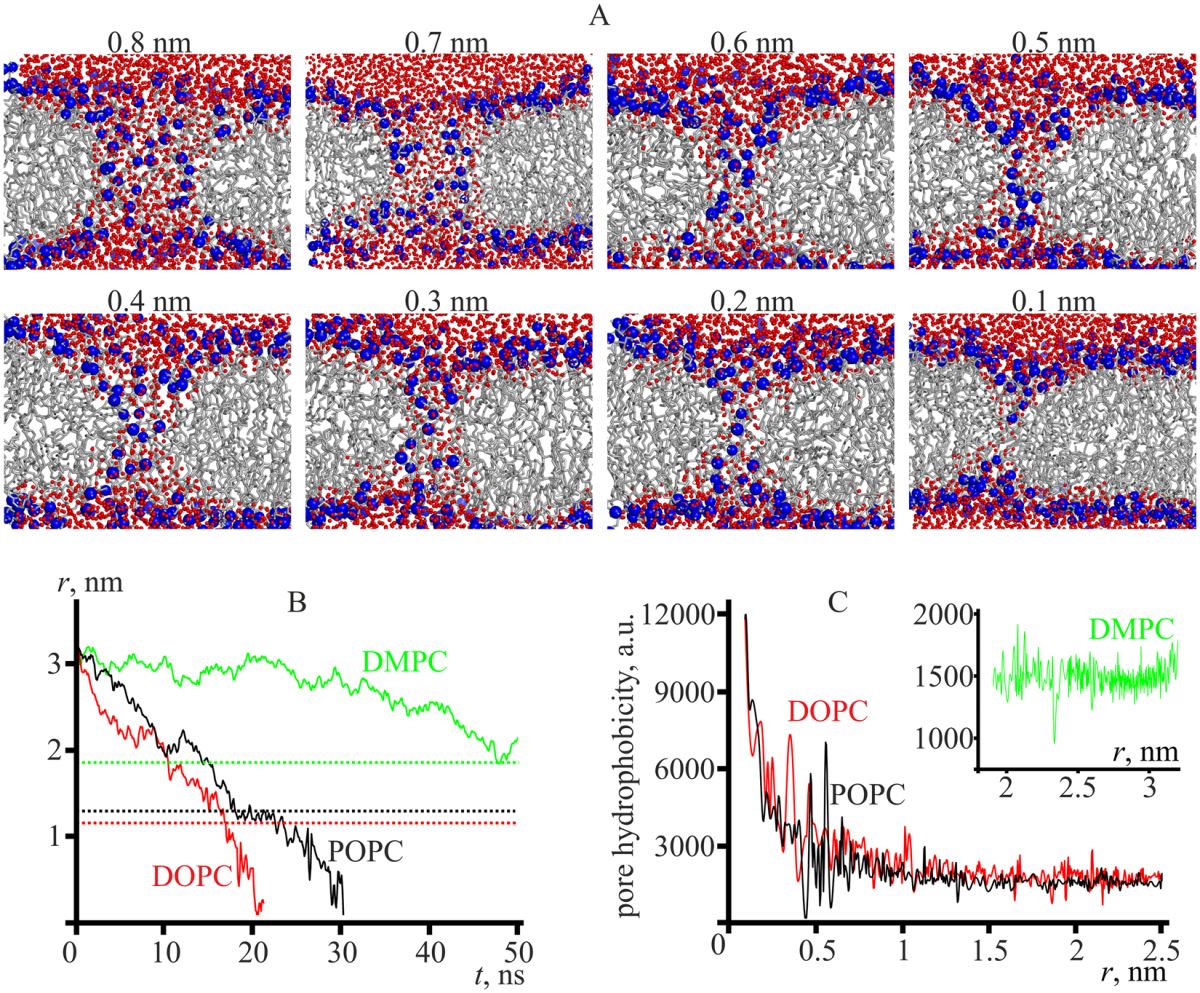
(**A**) Snapshots of POPC membrane (sideview) of a spontaneously closing pore obtained from molecular dynamics. Blue spheres represent polar atoms (phosphorus and nitrogen), red spheres represent water molecules. Lipid tails are shown as grey lines. Pore radius determined as described in the “Materials and Methods” Section is specified above every snapshot. (**B**) Time course of the pore radius in the molecular dynamics simulations of spontaneous pore closure. Red curve — DOPC membrane; black curve — POPC membrane, green curve — DMPC membrane. Dotted lines correspond to equilibrium pore radii, calculated in the framework of continuum theory. (**C**) Dependence of the hydrophobicity of the pore boundary on the pore lumen radius obtained in molecular dynamics simulations. Red curve — DOPC membrane; black curve — POPC membrane; inset, green curve — DMPC membrane. Each curve was averaged over two independent simulations.

We performed MD simulations of pore closure starting from a hydrophilic pore of the radius of about 3 nm. In Fig. 2A, the cross-section of POPC membrane by the plane passing through the pore axis is shown for different pore radii. As the lumen radius of the closing pore goes down, the density of polar heads of lipid molecules (blue spheres) gradually decreases at the edge of the pore, while the water molecules (red spheres) remain in the lumen. At a pore radius of 0.1 nm, a water-filled cylinder, the side surface of which is lined exclusively with hydrophobic chains of lipids (Fig. 2A, last frame), i.e. a hydrophobic defect, is formed. The water density inside the cylinder is lower than the density of the bulk water, indicating that the difference of water structure inside narrow cavities as compared to the bulk water should be taken into account.

Simulated time dependencies of the pore radius for DOPC, POPC, and DMPC membranes are shown in Fig. 2B. On the average, pores close faster in DOPC membranes (red curve), consistently with a somewhat higher line tension of the pore boundary compared to POPC membranes (black curve) based on experimental data^10^ and the continuum theory of elasticity estimates (Section “DOPC, POPC, and DMPC membranes”). The radius of the pore in DMPC membrane decreases very slowly (green curve). The radius reached about 2 nm and then fluctuated around this value. The slow decrease of the pore radius is consistent with the small value of line tension experimentally determined for DMPC (6.2 pN as compared to 11.5 pN for DOPC membrane under the same experimental conditions^45^), and calculated in the framework of continuum elasticity theory.

Molecular dynamic simulations of transition from hydrophilic pore to hydrophobic defect can provide insights into the mechanism of the process of formation of hydrophilic pore. Pore edge hydrophobicity was evaluated along the trajectory as described in the “Materials and Methods” section. The dependence *S*_*phob*_(*r*) is plotted in Fig. 2C. For DMPC membrane the pore remained hydrophilic till the end of the simulation trajectory (50 ns); the hydrophobicity of the pore is low and virtually constant (inset, green curve). For DOPC and POPC membranes (red and black curves) the hydrophobicity remained virtually constant down to the pore radius of about 0.8 nm: the entire pore surface was formed by polar heads of lipids. Upon further decrease of the radius, the edge hydrophobicity gradually increased with some surge of thermal noise amplitude at the pore lumen radius of *r*_*L*_ ∼ 0.5 nm (Fig. 2C). The overall increase of the hydrophobicity is consistent with the notion that hydrophobic defects are formed at small pore radii (Fig. 2A).

### Trajectory of pore formation via hydrophobic defect

**Figure 3:**
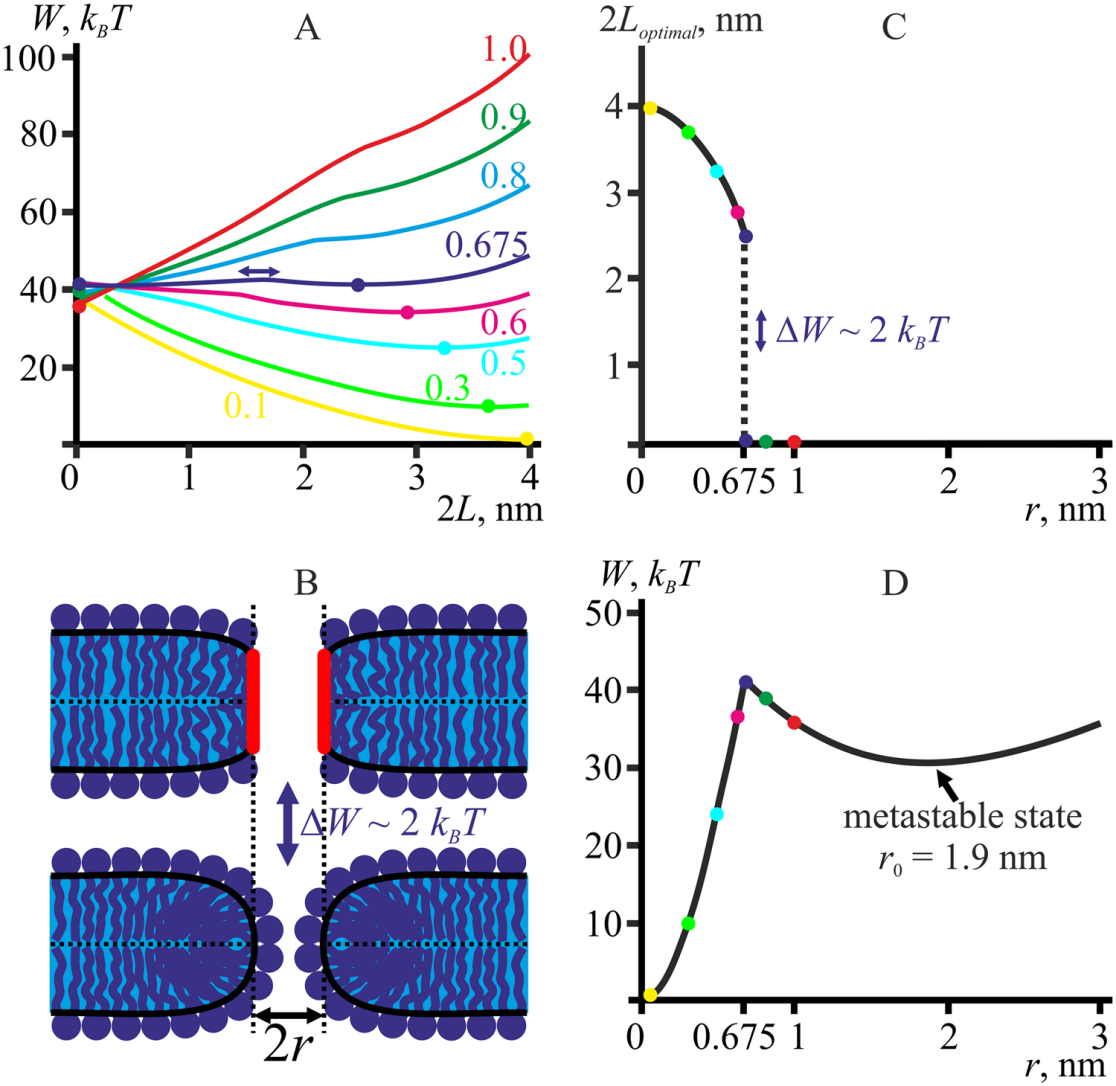
(**А**) Dependence of pore energy on the hydrophobic belt height, 2*L*, for the reference model lipid at different pore radii (specified near each curve in nanometers). The elastic parameters of the reference lipid are as follows: *B*_*m*_ = 8 *k*_*B*_ *T*, *h*_*m*_ = 2 nm, *K*_*A*_^*m*^ = 100 mN/m. The dependencies *W*(*L*) have minima, marked by color circles. For the radius *r* ∼ 0.675 nm (dark blue curve), pore energy *W*(*L*) has two minima with identical energies — at 2*L* = 0 and at 2*L* = 2.5 nm. (**B**) Schematic illustration of configurations, corresponding to two energy minima of the dark blue curve of the panel A: hydrophobic defect (2*L* = 2.5 nm, the hydrophobic belt surface is shown in red) and hydrophilic pore (2*L* = 0) of the same radius (*r* ∼ 0.675 nm). The states have equal energy, but substantially different cross-section of the pore lumen. The states are separated by low energy barrier of *ΔW* ∼ 2 *k*_*B*_ *T*, which implies high frequency of transitions between them. (**C**) Dependence of the optimal height of hydrophobic belt, 2*L*_*optimal*_, on the pore radius. *L*_*optimal*_ is obtained from positions of minima of the dependencies *W*(*L*), presented on the panel A and marked by color circles. (**D**) Dependence of optimal pore energy on the radius *r* for the reference model lipid. The optimal pore energy is the energy at the minima of the dependencies *W*(*L*), presented on the panel A and marked by color circles. The local minimum of the dependence *W*(*r*) at *r*_0_ ≈ 1.9 nm corresponds to a metastable state of the system.

The trajectory of pore formation via hydrophobic defect is analyzed for the reference model lipid and the hydrophobic interaction characteristic length of *ξ*_*h*_ = 1 nm. We vary the half-height of the hydrophobic belt, *L*, for a given pore radius and plot *W*(*L*) dependencies (Fig. 3A). At each radius the energy *W*(*L*) has a minimum; the minima are marked by color circles on each curve (Fig. 3A). Each minimum location determines the optimal height of the hydrophobic belt, 2*L*_*optimal*_ (horizontal axis), and the minimal energy value (vertical axis) at given pore radius. The optimal height of the hydrophobic belt depends on the pore radius. The dependence 2*L*_*optimal*_(*r*) is plotted in Fig. 3C. Determining the pore energy at the minimum (at 2*L* = 2*L*_*optimal*_) for each pore radius, we plot the optimal dependence of the pore energy *W*(*r*) (Fig. 3D). The color circles corresponding to the energy minima of the *W*(*L*) dependencies in Fig. 3A are marked on the curves of *W*(*r*) (Fig. 3D) and 2*L*_*optimal*_(*r*) (Fig. 3C). In Fig. 3A the curve *W*(*L*) corresponding to *r* ∼ 0.675 nm has two minima with the identical energies at the heights of the hydrophobic belt of 2*L* = 0 (hydrophilic pore) and 2*L* = 2.5 nm (hydrophobic defect), separated by an energy barrier of *ΔW* ∼ 2 *k*_*B*_ *T* (dark blue curve). The minima are marked by two dark blue circles. The relatively low height of the barrier implies a reasonably high frequency of transitions between the minima. It should be noted that for hydrophilic pore (*L* = 0) with the neutral surface radius of *r* ∼ 0.7 nm, the pore lumen is almost fully obstructed by polar heads of lipid molecules in the midplane (Fig. 3B). Indeed, the neutral surface goes approximately along the interface between the hydrophobic tails and polar heads of lipids, with the polar head size being about ∼0.7 nm^18^. For the hydrophobic belt height of 2*L* = 2.5 nm, there are no polar heads in the lumen and it has to be filled with water, making the membrane electrically conductive (Fig. 3B). Thus, if the characteristic time of change of the pore radius is much larger than the characteristic time of transitions between the two minima (hydrophilic pore at 2*L* = 0 and hydrophobic defect at 2*L* = 2.5 nm) separated by a small energy barrier, high frequency fluctuations of electric conductivity must be observed for the radius of conductive defect of about 0.7 nm. Such fluctuations, known as “flicker”, are indeed observed experimentally^12,46^.

The equal-energy states of the system at *r* ∼ 0.675 nm have substantially different hydrophobicity of the edge (hydrophilic pore at 2*L* = 0 and hydrophobic defect at 2*L* = 2.5 nm). Fast transitions between the states should result in high frequency noise of pore hydrophobicity. The noise was indeed observed in our MD simulations for pores of similar radii (see Fig. 2C). At smaller pore radii, the noise amplitude decreases and the average hydrophobicity of the pore boundary grows (Fig. 2C), as hydrophobic defects become a predominant state as predicted based on the continuum theory of elasticity (Fig. 3A).

At the radius *r* ∼ 0.675 nm the optimal energy *W*(*r*) has a maximum (Fig. 3D, dark blue circle), which determines the energy barrier of transition of hydrophobic defect (*r* < 0.675 nm) to hydrophilic pore (*r* > 0.675 nm). The energy of the hydrophilic pore has a local minimum at *r*_0_ ≈ 1.9 nm, which corresponds to a metastable state of the system (Fig. 3D). The energy barrier for transition of an intact bilayer (*r* = 0) into the metastable state (*r* = *r*_0_ ≈ 1.9 nm) is about 40 *k*_*B*_ *T*, the energy barrier for a reverse transition from the metastable state to the intact bilayer is about 10 *k*_*B*_ *T* for the reference model lipid.

### Pore energy and line tension in the framework of the continuous model

#### DOPC, POPC, and DMPC membranes

**Figure 4:**
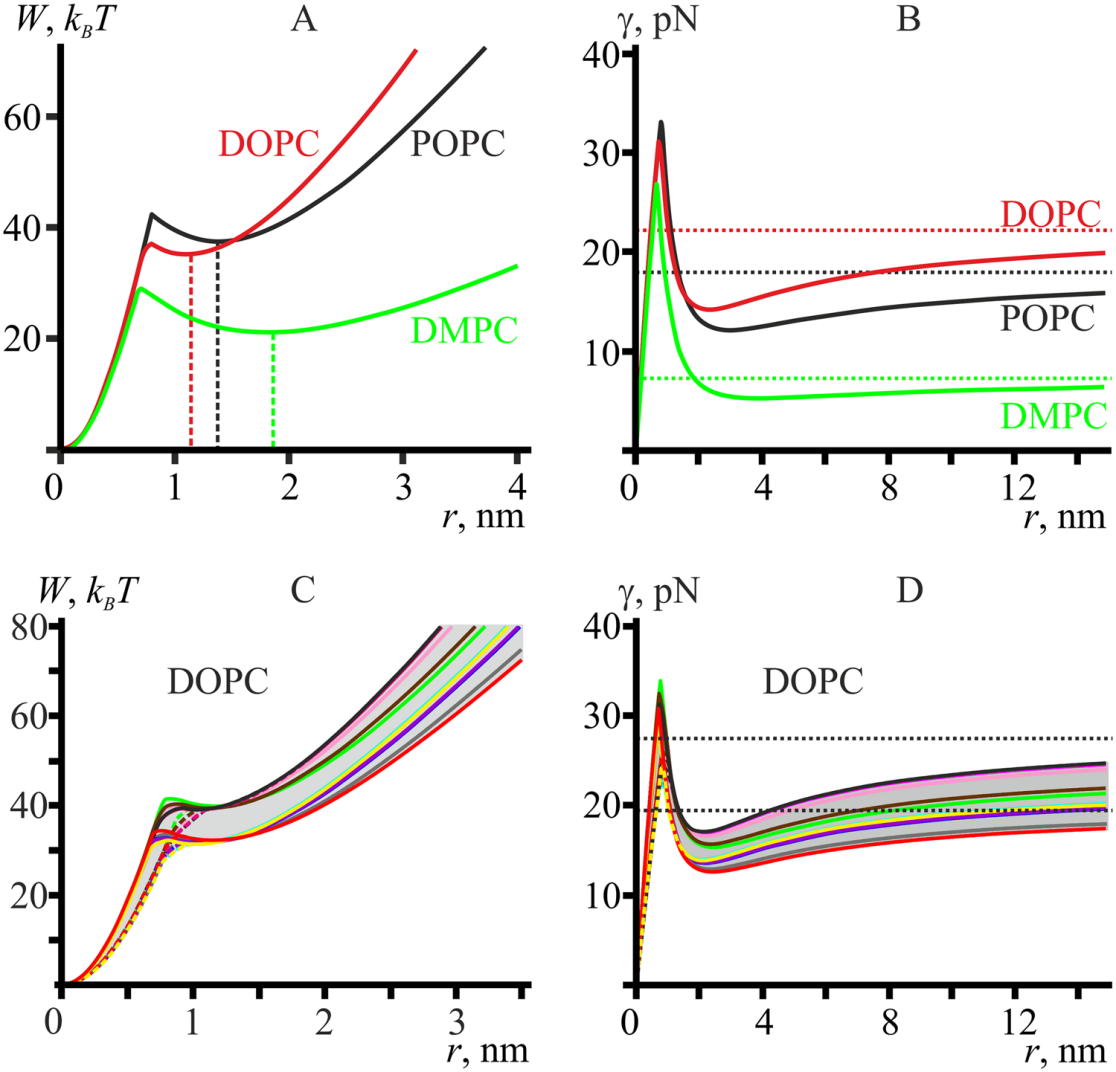
(**A**) Dependence of the pore energy on its radius for DOPC (red curve), POPC (black curve) and DMPC (green curve) membranes. Vertical dashed lines indicate the radius of a metastable pore for each membrane; the dashed lines correspond to those in Fig. 2B. (**B**) Dependence of the line tension on pore radius for DOPC (red curve), POPC (black curve) and DMPC (green curve) membranes. The dependencies were obtained by dividing corresponding *W*(*r*) from panel A by the pore perimeter, 2*πr*. Dashed horizontal lines correspond to asymptotic values of line tension *γ*_0_ at large (infinite) pore radius. (**C**) Dependencies of the pore energy on its radius for DOPC membrane for various experimentally determined sets of elastic parameters, built taking into account the confidence interval of the elastic parameters. (**D**) Dependencies of the line tension on the pore radius for DOPC membrane, obtained by dividing the energy *W*(*r*) (from panel C) by the pore perimeter, 2*πr*. Dashed horizontal lines correspond to minimal and maximal asymptotic values of line tension *γ*_0_ at large (infinite) pore radius.

Applying the same algorithm as described in the previous section for the reference model lipid, we obtain the dependencies of the pore energy on its radius for DOPC, POPC and DMPC membranes (Fig. 4A). All three membranes have metastable states at some pore radii, indicated by vertical dashed lines. The same lines are shown in Fig. 2B. For POPC membrane the dependence *r*(*t*) obtained from MD modeling has a plateau of about 5 ns duration (between 18 and 23 ns) at the radius indicated by the dashed line (Figs 2B, 4A). The plateau can be attributed to the system temporarily residing in the metastable state at *r* ∼ 1.4 nm determined by the local minimum of the pore energy of about 4.4 *k*_*B*_ *T* depth (Fig. 4A, black curve). For DOPC membrane, the depth of the local minimum is relatively small (less than 2 *k*_*B*_ *T*) (Fig. 4A, red curve), so in the MD simulations the system does not stay in the metastable state at *r* ∼ 1.1 nm long enough for the plateau to be distinguishable (Fig. 2B). However, the average slope of the MD dependence *r*(*t*) changes in the vicinity of the indicated radius at *t* ∼ 17 ns: the radius decreases faster for *r* < 1.1 nm (Fig. 2B, red curve), which is in agreement with a somewhat faster drop of the calculated pore energy as *r* → 0 (Fig. 4A, red curve). For POPC membrane, the MD trajectory at small radii is quite noisy, but it still allows concluding that the rate of the radius decrease becomes somewhat higher for *r* < 1.4 nm (Fig. 2B, black curve). For DMPC membrane the local minimum at *r* ∼ 1.9 nm is the deepest among the three lipids — about 8 *k*_*B*_ *T* (Fig. 4A, green curve). In the MD modeling, the pore slowly tends towards the minimum and fluctuates around it remaining hydrophilic until the end of the calculated trajectory (Fig. 2B, green curve).

Dividing the dependencies *W*(*r*) by the pore perimeter, 2*πr*, we obtained the corresponding dependencies of the line tension, *γ*(*r*) (Fig. 4B). The line tension is a non-monotonous function of the pore radius, saturating at large radii and having a minimum at the radius of *r* ∼ 2–4 nm with an abrupt increase at smaller values and final drop to zero as *r* → 0. Classical pore formation theory^47^ disregards this kind of dependence, as do some of the more recent theories^48^. However, the non-monotonous dependence of the line tension on pore radius was already obtained in early theoretical studies, in which toroidal shape of the pore boundary was postulated^14^. The non-monotonous dependence of the line tension on pore radius is caused by a non-monotonous dependence of energy on the radius (Fig. 4А). Dashed horizontal lines in Fig. 4B correspond to asymptotic values of line tension *γ*_0_ at large (infinite) pore radius. At large radii, *γ* tends to the limit of 22.2 pN for DOPC, 17.9 pN for POPC, and 7.2 pN for DMPC membranes (Fig. 4B). The minimal values of the line tension for hydrophilic pore are 14.2 pN at *r* ∼ 2.3 nm for DOPC (Fig. 4B, red curve); 12.1 pN at *r* ∼ 3 nm for POPC (Fig. 4B, black curve); and 5.3 pN at *r* ∼ 4 nm for DMPC (Fig. 4B, green curve). The curves *γ*(*r*) for DOPC and POPC almost coincide for *r* < 2 nm, while they differ substantially for larger radii (Fig. 4B).

For POPC there is no reliable statistics on the experimentally determined values of line tension, to the best of our knowledge. T. Portet and R. Dimova^10^ measured the line tension and reviewed the data for egg PC; the line tension is shown to vary in the range of 8.6-42 pN. However, egg PC is a mixture of lipids with different length of hydrocarbon chains and different degree of saturation. According to Avanti^49^, POPC content in eggPC is about 60%.

To the best of our knowledge, for DMPC there are no statistically reliable data on the value of the line tension either. However, E. Evans and co-workers present the data for diC13:0 lipid, which is quite similar to DMPC (diC14:0); the reported value is 6.2 pN for pores of the radius of a few nanometers^45^. This value is slightly higher than our calculated minimal value for small radii (∼5.2  pN), but in the work ref.^45^ the pores are formed by application of the lateral tension, which, as we demonstrate in the accompanying paper^19^, should be expected to increase the line tension somewhat. In the work ref.^50^ the authors experimentally determined the line tension for large (∼1 μm) pores formed in 1,2-dilauroyl-sn-glycero-3-phosphocholine (DLPC), and DPPC. The corresponding values are 2.5 ± 0.3 pN; 9.5 ± 1.0 pN. It is found that each methylene group of lipid acyl chain contributes 0.7 ± 0.2 pN to the line tension. Thus, for DMPC one can obtain 2.5 + 4·0.7 = 5.3 ± 1.1 pN if add the contribution of four methylene groups to the line tension of DLPC, or 9.5 − 4·0.7 = 6.7 ± 1.8 pN if subtract the contribution of four methylene groups from the line tension for DPPC. Our calculated value for large pores (*γ*_0_ ∼ 7.2 pN) lies close to the upper boundary of the phenomenologically derived range^50^.

In order to calculate the energy and the line tension of the pore, we utilized experimentally determined values of the elastic parameters. These values are known with finite uncertainties, which are presented for DOPC membranes in the section “System parameters”. We combined different limiting values of the elastic parameters — the upper and lower limits of the experimental confidence interval — to distinct sets and calculated the dependencies of the pore energy, *W*(*r*) (Fig. 4C), and of the line tension *γ*(*r*) (Fig. 4D) for DOPC membrane. The curves generated for different sets of the elastic parameters form a spectrum of possible dependencies *W*(*r*) and *γ*(*r*) (Fig. 4C,D, shown in light grey). The depth of the local energy minimum, *W*(*r*), varies in the range 0 to 2 *k*_*B*_ *T* (Fig. 4C). The asymptotic value of the line tension for large (infinite) pore radius varies in the range *γ*_0_ ∼ 19.5–27.4 pN (Fig. 4D, dashed lines); the minimal value of the line tension for hydrophilic pore is 12.5 pN, which is achieved at the pore radius of 2.4 nm (Fig. 4D). For DOPC membranes, the results of our calculations along with the experimentally determined values of the line tension are summarized in Table 1.

**Table 1:** Line tensions of pore edge in DOPC membrane.

*γ*, pN	Our average value	Our limiting values	Experimentally determined values						
Small pores	14.2	12.5–17.0					11.5^45^	10.5^51^	11.6^52^
Large pores	22.2	19.5–27.4	6.9 or 20.7^53^	11.4^50^	27.7^10^	18^54^			

Our calculated values are presented on Fig. 4B (average values) and Fig. 4D (limiting values). Small pores correspond to the local minimum of the line tension dependence *γ*(*r*); large pores correspond to *r* → ∞.

From Table 1 it is seen that the experimentally determined values of the line tension depend on the measurement method, or, more precisely, on the size of the pore for which the line tension was obtained. For large macroscopic pores (*r* ≥ 1 μm) the experimental values of the line tension are of the order of 20 pN as obtained in three works^10,53,54^; our calculated values are thus in good agreement with these experimental data. In the work ref.^53^ two values of the line tension are obtained for DOPC: 6.9 pN for DOPC from Sigma, and 20.7 pN for DOPC from Avanti and from Fluka. The authors attribute the lower value to the presence of small amount of impurities, treating the higher value as being more reliable. In the work ref.^50^ the reported value of the line tension for large pores is 11.4 pN, which notably deviates from ∼20 pN. However, the experimental conditions of this work substantially differ from those of the works refs^10,53,54^ by complete absence of sugars and presence of 11 mM MgCl_2_ in the bathing solution. Bivalent ions Mg^2+^ can cause clustering of lipids, which can lead to alteration of membrane elastic properties, especially, spontaneous curvature of its composing monolayers. Clustered lipids can be considered as an impurity, which may heterogeneously distribute between pore edge and the bulk membrane, thus leading to decrease of the effective line tension^53^.

**Figure 5:**
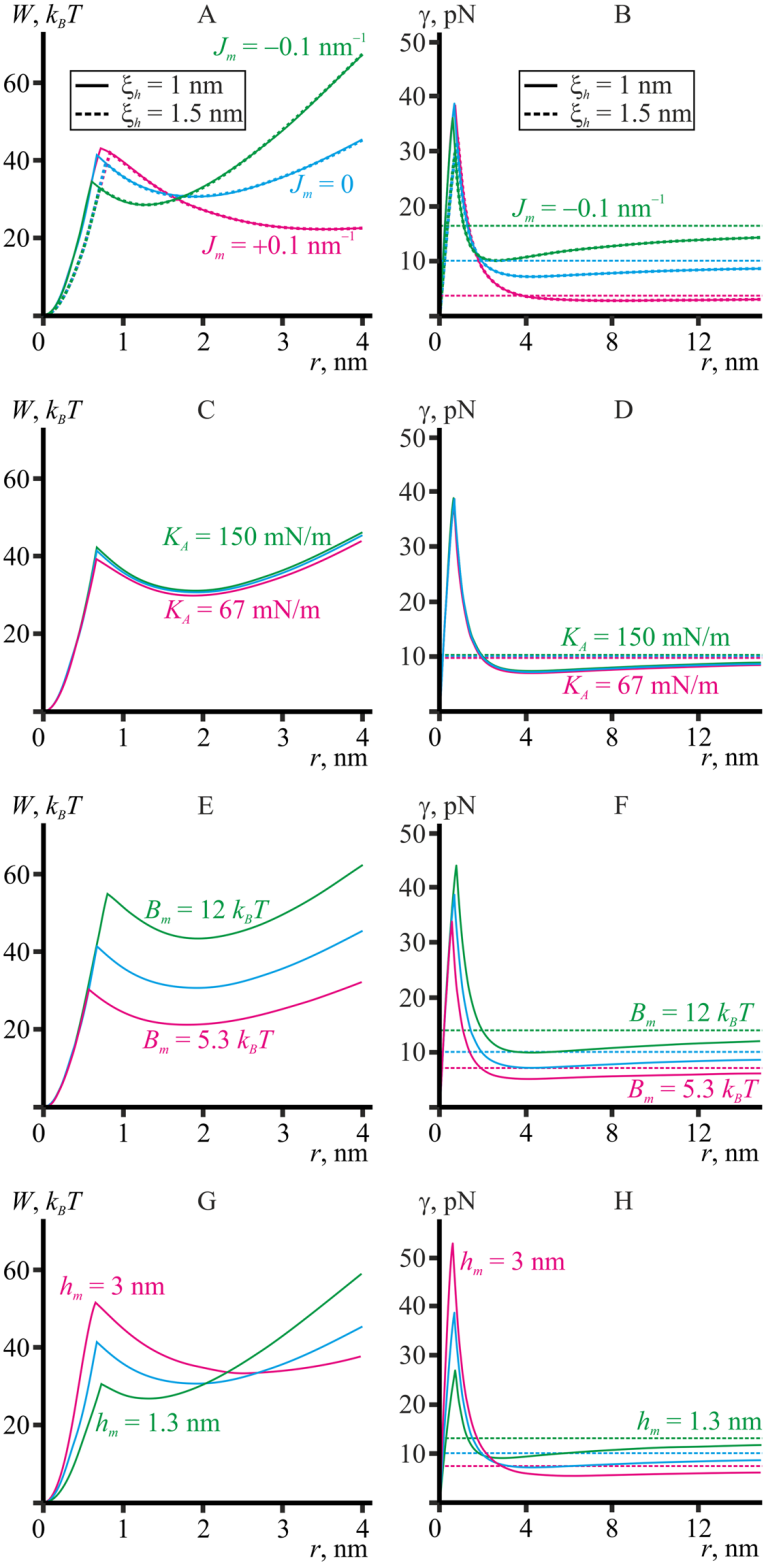
Dependence of energy (**А**,**C**,**E**,**G**) and line tension (**B**,**D**,**F**,**H**) of the pore on its radius *r* obtained based on the continuum theory of elasticity for model lipid bilayers. Blue curves in all plots correspond to the reference model lipid (*B*_*m*_ = 8 *k*_*B*_ *T*, *K*_*A*_^*m*^ = 100 mN/m, *h*_*m*_ = 2 nm, *J*_0_ = 0). (**A**,**B**) Spontaneous curvatures *J*_*m*_ = +0.1 nm^–1^ (magenta curves) and *J*_*m*_ = –0.1 nm^–1^ (green curves); (**C**,**D**) splay moduli *B*_*m*_ = 5.3 *k*_*B*_ *T* (magenta curves) and *B*_*m*_ = 12 *k*_*B*_ *T* (green curves); (**E**,**F**) lateral stretch/compression moduli *K*_*A*_ = 67 mN/m (magenta curves) and *K*_*A*_ = 150 mN/m (green curves); (**G**,**H**) thicknesses of the hydrophobic part of monolayer *h*_*m*_ = 3 nm (magenta curves) and *h*_*m*_ = 1.3 nm (green curves). All parameters except indicated are taken the same as of the reference model lipid. Dashed horizontal lines on the panels B,D,F,H correspond to asymptotic values of line tension *γ*_0_ at large (infinite) pore radius.

For small pores, the radius of which is of the order of a few nanometers, the experimental values of the line tension lie in a narrow range of 10-12 pN^45,51,52^. These values are somewhat beyond the range of the *γ*(*r*) curves presented in Fig. 4D. We hypothesize that this can be explained by the elastic parameters utilized in our calculations being determined in different experimental systems and under different conditions. More specifically, the line tension and the elastic moduli of splay and lateral stretch/compression are determined in the refs^10,39,45^ on giant unilamellar vesicles (GUV) in sugar-containing solution with small amount of salt (1 mM NaCl in ref.^10^), while the spontaneous curvatures are measured in the refs^18,40^ on monolayers in inverted hexagonal phase lacking sugars and salt in the aqueous bath. It is shown that the presence of 200 mM of sugar in the bathing solutions can cause a decrease of membrane splay rigidity by a factor of two or more^55^; such concentrations are characteristic for experiments with GUVs. It is reasonable to assume that sugars can also affect the spontaneous curvature, although such kind of influence had not been quantitatively analyzed. Thus, the spontaneous curvature of the monolayers constituting the membrane of GUVs is not exactly known, while, as we demonstrate below (Fig. 5A,B), the spontaneous curvature strongly affects the pore line tension.

Formally using Eq. (1), the critical radius of pores formed in DOPC membranes by application of the lateral tension σ_0_ can be estimated as:

23 $${r}^{\ast }=\frac{\gamma }{{\sigma }_{0}}\approx \frac{11\,{\rm{pN}}}{7\,\mathrm{mN}/m}\approx 1.6\,\mathrm{nm},$$

where a typical value of the lateral tension σ_0_ = 7 mN/m^45^ and the line tension measured for subcritical pores, *γ* ∼ 11 pN (Table 1), were assumed. The corresponding line tension calculated for such a pore radius in the framework of the continuum theory is substantially higher, *γ* ∼ 25 pN (Fig. 4B, red curve). However, the Eq. (1) is derived for infinitely thin films lacking any internal structure^6^. If pore radius is equal to or smaller than the membrane thickness, the membrane cannot be considered as an infinitely thin film. Besides, during pore formation, orientation of lipid molecules changes from vertical in the intact bilayer to horizontal in a hydrophilic pore (Fig. 1A), i.e. crucial change of membrane structure takes place. This also means that the membrane in this process cannot be considered as a structureless film. The equations used in the recent papers on pore formation in lipid membranes are modified and generalized by introduction of various pre-pore states^45,52,56^, although still maintaining the assumption that the line tension is constant, at least for some range of radii. At the same time, if one divides the presented dependencies^45,52,56^ of pore energy on pore radius, *W*(*r*) (for zero lateral tension), by the pore perimeter (2*πr*), the resulting line tension *γ* = *W*(*r*)/(2*πr*) will be far from constant if considered in the whole range of pore radii. Thus, for practical applications generalization of the Eq. (1) is unavoidable because Derjaguin model is very rough approximation for the dependence of the pore energy on the pore radius, failing to yield a satisfactory explanation of the experimentally observed pore formation kinetics^45,52,56^. This is because membranes have a finite thickness^39,42,45^ and possesses internal structure^20^, which in our continuum approach is modeled by introduction of a vector field of directors, **n**, characterizing the average orientation of lipid molecules. The critical radius should thus be obtained from the position of the maximum of the pore energy *W*(*r*) at a given lateral tension. In the accompanying paper^19^, the calculated position of the maximum for DOPC is *r* ^*^ ∼ 4 nm for the lateral tension ∼7 mN/m, thus resulting in substantially smaller (than 25 pN) values of the calculated line tension of subcritical pores (Fig. 4B).

#### Model lipid membranes

In Fig. 5, the energy and line tension calculated based on the elasticity theory for continuous medium are plotted as functions of the pore radius for different model lipids. Figure 5B,D,F,H show that line tension is a non-monotonous function of the pore radius, saturating at large radii and having a local minimum at the radius of *r* ∼ 3–8 nm with abrupt increase at smaller values (*r* ∼ 0.6–0.8 nm) and final drop to zero on the stage of hydrophobic defect as *r* → 0. The non-monotonous dependence of the line tension on pore radius is caused by a non-monotonous dependence of energy on the radius (Fig. 5А,C,E,G).

Trajectories of pore formation via hydrophobic defect for model lipids with different spontaneous curvatures are shown in the Fig. 5A for characteristic lengths of hydrophobic interaction of *ξ*_*h*_ = 1 nm and *ξ*_*h*_ = 1.5 nm. The energy barrier to pore formation from a hydrophobic defect decreases at negative spontaneous curvatures of the membrane monolayers and increases at positive spontaneous curvatures. This behavior is the opposite of that for the line tension (Fig. 5B). Change of the model lipid spontaneous curvature from −0.1 nm^−1^ to +0.1 nm^−1^ causes the energy barrier to increase by ~10 *k*_*B*_ *T* (from 33 *k*_*B*_ *T* to 43 *k*_*B*_ *T*). The energy barrier for the reverse transition (hydrophilic pore closure) is increasing at higher spontaneous curvatures, being ~5 *k*_*B*_ *T* at the *J*_*m*_ = −0.1 nm^−1^; and ∼20 *k*_*B*_ *T* at *J*_*m*_ = +0.1 nm^−1^ for the model lipid. The barriers are weakly sensitive to changes of the characteristic length of hydrophobic interactions, *ξ*_*h*_ (see Fig. 5A). The radius of transition from a hydrophobic defect to a hydrophilic pore does depend on it, increasing by about 0.1 nm when *ξ*_*h*_ changes from 1 to 1.5 nm. Overall, the radius of transition to hydrophilic pore increases with the increase of spontaneous curvatures, elastic moduli and characteristic length of hydrophobic interactions. The absolute changes of the radius are, however, minute: they vary within a narrow band with the width of about a few angstroms for the entire spectrum of parameters and lipid species considered (Fig. 5A), as opposed to line tension differing by the factor of up to 4.5 (see Fig. 5B). Hereafter, the results of calculations are presented only for the hydrophobic interaction length of *ξ*_*h*_ = 1 nm.

Major portion of elastic energy of a hydrophilic pore is stored in splay deformation. This follows from comparison of characteristic energy densities of deformations. Tilt and lateral stretch/compression moduli have the dimensionality of energy per unit area, while the dimensionality of splay modulus is energy. To enable comparison, *B*_*m*_ should be divided by square of the characteristic length of lipid monolayer that scales together with the *B*_*m*_, e.g., its thickness *h*_*m*_ = 2 nm. Thus, the characteristic energy density of splay is *B*_*m*_/*h*_*m*_^2^ = 8 mN/m; whereas for tilt and lateral stretch/compression they are *K*_*t*_ = 40 mN/m and *K*_*A*_^*m*^ = 100 mN/m, respectively. Thus, splay is the softest deformational mode storing the major part of the elastic energy. This is illustrated by Fig. 5E,F, where the variation of the splay moduli by the factor 1.5 results in change of the line tension by almost the same factor. Lateral stretch/compression is the most rigid deformational mode; hence, there is almost no change in the total elastic energy and the line tension of the pore (Fig. 5C,D) upon variation of the lateral stretch/compression modulus *K*_*A*_ by the factor of 1.5. The characteristic energy density of tilt deformation is intermediate. However, we do not vary the tilt modulus, since, according to estimation of the work ref.^20^, *K*_*t*_ should be approximately equal to the surface tension at lipid tails/water interface, i.e. should not strongly depend on lipid composition of the membrane.

Splay deformation is determined by the sum of the principal curvatures of the neutral surface less the monolayer spontaneous curvature^22^. The curvature of the pore boundary surface has a positive meridional and a negative equatorial components, the meridional one being nearly constant and equal to 1/*h*, whereas the equatorial curvature strongly depends on the radius, being of the order of −1/*r*. For the pore radius of *r* ∼ *h* these components practically annihilate, 1/*h* − 1/*r* ≈ 0, causing an energy minimum to occur at the radius of a few nanometers. At zero spontaneous curvature (Figs 3D and 5A blue curve), the energy minimum is attained almost exactly at *r* = *h*. Negative spontaneous curvature shifts the minimum towards smaller pore radii (Fig. 5А, green curve); positive curvature causes the opposite shift (Fig. 5А, magenta curve). The line tension minimum is at the radius of a few nanometers. Line tension and energy of the pore boundary notably depend on the spontaneous curvature of the membrane monolayer (Fig. 5A,B). At large pore radii, the deformation energy of the pore boundary is determined by large positive meridional curvature ∼1/*h*. Thus, the boundary energy and the asymptotic value of line tension *γ*_0_ (for *r* → ∞) decrease in case of positive spontaneous curvature (Fig. 5B, magenta curve), and increase if it is negative (Fig. 5B, green curve). At small radii, i.e. at *r* < 1.5 nm for the selected values of parameters, negative equatorial curvature becomes prevalent, causing inversion of the pore energy dependence on the spontaneous curvature: positive spontaneous curvature increases the energy of the boundary and vice versa (see magenta and green curves on Fig. 5А). At small pore radii, the pore boundary line tensions in the membranes made of lipids with different spontaneous curvature converge (see Fig. 5B): the line tension difference between the model lipid with positive and negative spontaneous curvatures tends to ~12.5 pN at infinite pore radius (compare green and magenta dashed horizontal lines on Fig. 5B), but vanish at the pore radius of ∼2 nm (compare green and magenta curves on Fig. 5B). It is also in qualitative agreement with the experimental results presented in ref.^10^ and in Table 1: the line tensions measured for large radius pores in the membranes of different composition differ much more significantly than those measured on small pores.

The energy barrier of the transition from hydrophobic defect to hydrophilic pore depends on spontaneous curvature of the monolayer. The energy of the hydrophilic pore depends on spontaneous curvature (Fig. 5A), because this energy is largely determined by splay deformations. With the gradual transformation of the hydrophilic pore into a hydrophobic defect, we replace the highly deformed equatorial part of the pore with a hydrophobic cylinder whose interfacial tension depends on the radius. This process becomes favorable when the elastic energy of the equatorial pore region is greater than the energy of contact of the hydrophobic cylinder of the same radius with water. The elastic energy of the equatorial region depends on the magnitude of the spontaneous curvature of the monolayer. Thus, the radius and barrier of the transition between hydrophilic pore and hydrophobic defect should also depend on the spontaneous curvature of the monolayer (Fig. 5A).

The predominance of the positive meridional curvature ∼1/*h* at large pore radius results in about 1.5 times smaller line tension of the pore in a thicker bilayer (*h*_*m*_ = 3 nm) compared to the reference bilayer (Fig. 5H, magenta curve), while the line tension of the pore in a thinner bilayer (*h*_*m*_ = 1.3 nm) is about 1.5 times larger than for the reference bilayer (Fig. 5H, green curve). The pore energy minimum at the radius of *r* ≈ *h* is clearly illustrated by Fig. 5G. Note that variation of the elastic moduli does not change the energy minimum position (Fig. 5C,E).

Table 2 summarizes absolute and relative contributions to the energy of the hydrophobic defect and hydrophilic pore along the optimal trajectory of pore formation for the reference model lipid. On the stage of the hydrophobic defect (*r* < 0.675 nm) the major contribution to the energy is from the hydrophobic belt. When the pore radius exceeds about 0.675 nm, the hydrophobic belt vanishes, and the pore becomes hydrophilic. The major part of the elastic energy of hydrophilic pore is stored in splay deformations (Table 2).

**Table 2:** Contributions of different deformational modes to the total energy of the pore edge in the membrane made of the reference model lipid.

Pore radius, *r*, nm	0.2	0.4	0.6	1	2	5	10	15	200
**Splay energy**, ***k***_***B***_ ***T*** **/%**	0.03 0.68%	0.46 2.74%	2.45 7.18%	32.4 90.4%	26.5 86.3%	45.5 80.0%	103 80.9%	166 81.6%	2622 83.4%
**Tilt energy**, ***k***_***B***_ ***T*** **/%**	0.06 1.27%	0.57 3.45%	2.54 7.43%	1.70 4.8%	2.70 8.8%	7.90 14.0%	16.9 13.3%	25.8 12.7%	356 11.3%
**Stretching energy**, ***k***_***B***_ ***T*** **/%**	0.01 0.25%	0.18 1.07%	1.11 3.25%	1.70 4.8%	1.50 4.9%	3.50 6.0%	7.40 5.8%	11.6 5.7%	168 5.3%
**Hydrophobic energy**, ***k***_***B***_ ***T*** **/%**	4.33 97.8%	15.5 92.7%	28.1 82.2%	0 0%	0 0%	0 0%	0 0%	0 0%	0 0%
**Total energy**, ***k***_***B***_ ***T***	4.41	16.6	34.1	35.8	30.7	56.9	127	204	3145
**Line tension, pN**	14.0	26.4	36.2	22.8	9.77	7.24	8.09	8.66	10.0

**Figure 6:**
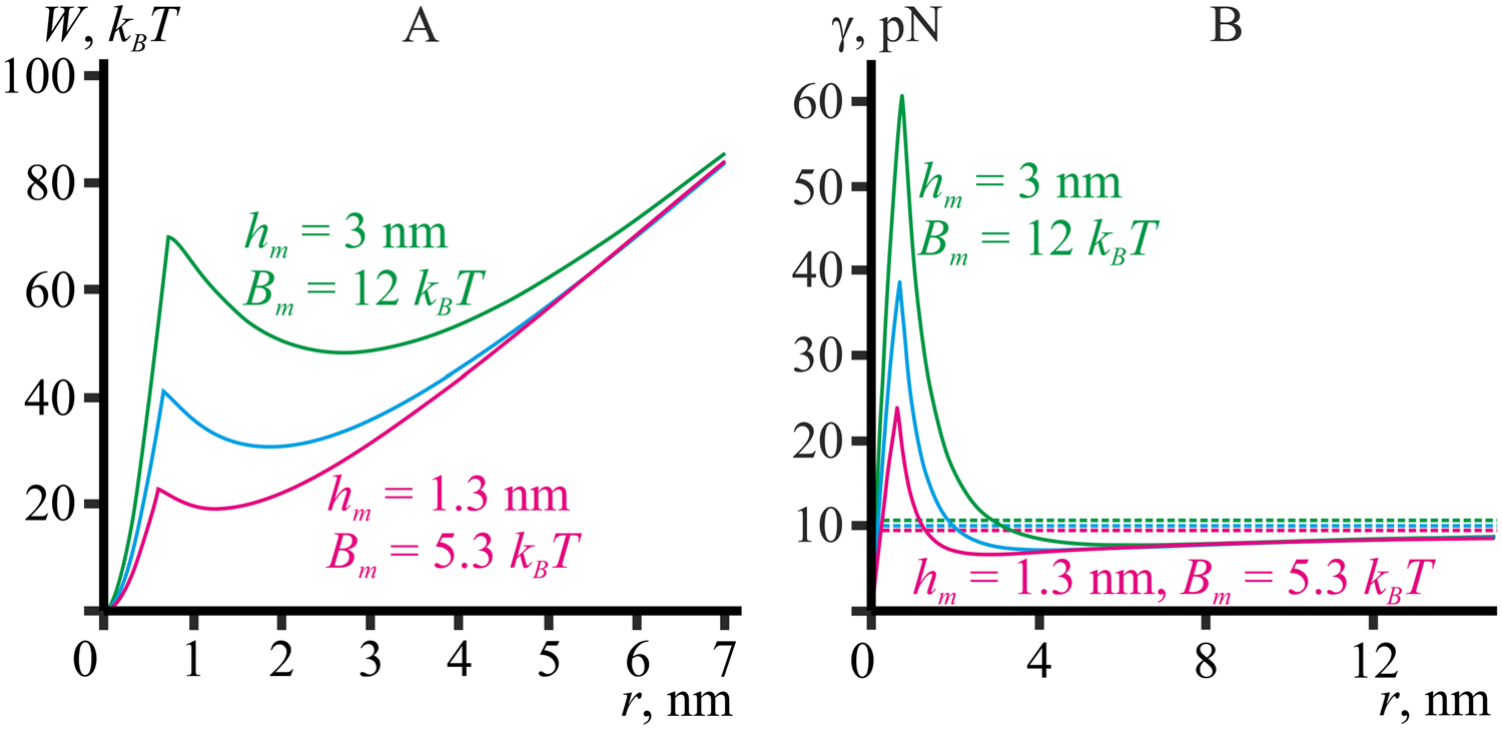
Dependence of energy (**А**) and line tension (**B**) of the pore on its radius *r* obtained based on the continuum theory of elasticity for the model lipid bilayers. Blue curves correspond to the reference model lipid (*B*_*m*_ = 8 *k*_*B*_ *T*, *K*_*A*_^*m*^ = 100 mN/m, *h*_*m*_ = 2 nm, *J*_0_ = 0); green curves — to model lipid with *h*_*m*_ = 3 nm, *B*_*m*_ = 12 *k*_*B*_ *T*; magenta curves — to model lipid with *h*_*m*_ = 1.3 nm, *B*_*m*_ = 5.3 *k*_*B*_ *T*. All other parameters are the same as for the reference model lipid. Dashed horizontal lines on the panel B correspond to asymptotic values of line tension *γ*_0_ at large (infinite) pore radius.

From Fig. 5E–H it is seen that an increase of the splay modulus *B*_*m*_ by the factor 1.5 and a decrease of the monolayer thickness *h*_*m*_ by the same factor result in almost equal change of the limiting line tension *γ*_0_ (from 10.0 pN to 13.2–13.9 pN). Besides, a decrease of the splay modulus and an increase of the monolayer thickness, both by the factor 1.5, result in almost equal decrease of the limiting line tension *γ*_0_ (from 10.0 pN to 7.1–7.4 pN). This observation prompts an assumption that for zero spontaneous curvature the limiting value of the line tension is proportional to the ratio *γ*_0_ ∼ *B*_*m*_/*h*_*m*_. To test the hypothesis, we considered pores in membranes made of three model lipids with almost the same ratio of *B*_*m*_/*h*_*m*_: i) the reference model lipid, *B*_*m*_ = 8 *k*_*B*_ *T*, *h*_*m*_ = 2 nm (Fig. 6, blue curves); ii) model lipid with *B*_*m*_ = 5.3 *k*_*B*_ *T*, *h*_*m*_ = 1.3 nm (Fig. 6, magenta curves); iii) model lipid with *B*_*m*_ = 12 *k*_*B*_ *T*, *h*_*m*_ = 3 nm (Fig. 6, green curves).

According to Fig. 6, both the energies and the line tensions of the pores almost coincide at large (infinite) pore radius. However, at small radii the curves differ substantially. In particular, the energy minimum (Fig. 6A) still remains at the position *r* ∼ *h*_*m*_, which is different for the model lipids considered. Combining high splay modulus and increased monolayer thickness (*B*_*m*_ = 12 *k*_*B*_ *T*, *h*_*m*_ = 3 nm) results in unaltered limiting line tension *γ*_0_, while the transition barriers grow ultimately, to 70 *k*_*B*_ *T* for forming the hydrophilic pore from the hydrophobic defect and to 21.5 to close the hydrophilic pore (Fig. 6A, green curve). On the contrary, low splay modulus and small monolayer thickness (*B*_*m*_ = 5.3 *k*_*B*_ *T*, *h*_*m*_ = 1.3 nm) lead to abrupt drop of the transition barriers to 22.5 *k*_*B*_ *T* (formation of hydrophilic pore from hydrophobic defect) and 3.5 *k*_*B*_ *T* (closing of the hydrophilic pore), respectively, at almost constant value of the limiting line tension *γ*_0_ (Fig. 6A, magenta curve). In principle, one can carefully adjust lipid splay modulus and monolayer thickness by choosing lipids with varying length of hydrocarbon tails and degree of saturation. From the results illustrated by Fig. 6 we can predict that if two lipids have the same *B*/*h* ratio (and zero spontaneous curvature), they should have the same line tension *γ*_0_ measured on large pores (*r* → ∞) (Fig. 6B), whereas the line tension of small pores formed in the membranes made of these lipids can differ substantially (Fig. 6B).

## Discussion

We have developed a model of hydrophilic pore formation from an intact bilayer through a hydrophobic defect. The energy of the pore boundary was calculated with the use of liquid crystal elasticity theory adapted to lipid membranes^20^. Since this theory was developed for small deformations, it is not directly applicable to pore boundary. However, by decomposing the boundary into smaller parts and defining deformations within each part as deviations from its own reference surface the deformations can be made small enough to allow decreasing the artificial overestimation of the calculated elastic energy. The main criterion of adequacy of the assumptions made, including the membrane decomposition into segments, is agreement of the results with the experimental data.

One of the key parameters of membrane stability with respect to pore formation, the line tension of the pore boundary, was found to be a function of the elastic parameters of the membrane and the pore radius (Figs 4, 5). Therefore, depending on the experimental technique, different values of line tension can be obtained for membranes of identical compositions, and equal values of line tension can be measured for pores in substantially different membranes.

We used pore radius in the equatorial plane *r* and half-height of the hydrophobic belt *L* as generalized coordinates defining pore state. Minimizing the energy with respect to *L* for each fixed *r*, we obtained the optimal trajectory of pore formation. In our MD trajectories we observed a state corresponding to the hydrophobic defect, the walls of which are partly formed by lipid tails, just before complete closure of pre-formed pores (Fig. 2A). In a coarse-grain (CG) MD modeling, T.V. Tolpekina and co-workers^57^ observed pore formation through local decrease of lipid head density, rather than through hydrophobic defects. However, in the CG representation water molecules are considered as particles equivalent to polar lipid heads, i.e. they are relatively large. This can alter the optimal trajectory of pore formation due to geometrical restraints: at small radii, the large water particles do not fit the narrow channel, and energetically favorable hydrophilic pore can be formed only when the channel radius becomes large enough to accommodate these particles. Later on, absence of the hydrophobic defect on the trajectory of pore formation was reported based on all-atom MD modeling^58^. However, in this work the size of the simulation box is such that the membrane is subjected to large effective lateral pressure of about 10 mN/m. This can lead to obstruction of a narrow channel by lipids that maximize lateral scatter due to insufficiency of the area per molecule.

Clearly, hydrophobic belt with a precisely defined boundary (Fig. 1B) is only a convenient model. In our MD modeling of pore closure, we merely observe the increase of the average hydrophobicity of the pore edge (Fig. 2C). The hydrophobic tails occur on the pore boundary surface randomly (Fig. 2A) and accordingly create local changes of hydrophobic and elastic properties, rather than forming a geometrically perfect cylindrical belt. However, following an approach similar to Gibbs method of excess values^59^, we can artificially divide the averaged mixed configuration (monolayer with somewhat reduced density of polar lipid heads) into two pure configurations (purely hydrophobic belt and vertical monolayer with equilibrium density of polar lipid heads), homogeneous up to their boundary — circular lines {*r*, ±*L*}, thus having zero-area boundary layer. In this sense, the local decrease of lipid heads density, obtained as the intermediate structure of pore formation in works refs^57,58^, is somewhat similar to the hydrophobic defect postulated in the present work and observed in our MD (Fig. 2A). Another reason for introducing a hydrophobic defect as an intermediate stage of pore formation is that the initial state of intact bilayer and the final state of hydrophilic pore can thus be continuously and seamlessly connected. In earlier works^12,13^ the pre-pore state was represented by a simpler model of a hydrophobic cylinder spanning the entire membrane thickness of 2 *h*. However, such a construct cannot be continuously transformed into a hydrophilic pore without allowing variations of the cylinder height and deformations of the membrane around the cylinder, which essentially is how we obtained the hydrophobic defects.

In the MD simulations, we deliberately made no attempts to calculate the pore energy as a function of its radius because a simulation in a thus restricted phase space would be misleading, i.e. the predicted phase trajectory of pore formation would be exclusively determined by the arbitrary choice of coordinate and have no relevance to the actual phase trajectory. Effectively, it would mean collapsing a 2D phase portrait to its 1D projection^17,57,58^. According to our results, two coordinates, *r* and *L*, define the state of the system at least for small pore radii. To avoid possible artifacts originating from the incorrect choice of the pore formation coordinate, we simply allowed the pre-formed pore to close spontaneously. Thus, we cannot determine the system energy along the trajectory (it is impossible for spontaneous closing), but we were able to analyze the intermediate structures formed along the trajectory.

One of the outputs of our MD modeling is the dependence of the pore radius on time, *r*(*t*) (Fig. 2B). In the work ref.^60^, the following equation was obtained for the rate of spontaneous pore closure:

24 $$\frac{dr}{dt}=-\frac{\gamma }{4\eta h},$$

where *η* is the dynamic viscosity of the membrane and *h* is the monolayer thickness. At the first glance, the dependencies of *r*(*t*) obtained for DOPC, POPC and DMPC by MD are nearly linear (Fig. 2B) prompting a conclusion that the line tension should be constant and independent on the pore radius:

25 $$\frac{dr}{dt}=const=-\frac{\gamma }{4\eta h}.$$

However, the relation (24) inherently relies on the assumption that the line tension is constant^60^. In practice, this equation is only used for describing the evolution of optically visible micron-sized pores^49,53,60^, where the assumption of line tension constancy holds true with a high accuracy. The relation is derived by equating the viscous force to the effective mechanical force driving changes of the pore radius. For the case of radius-dependent line tension, the equation transforms into the following:

26 $$\frac{dr}{dt}=-\frac{1}{8\pi \eta h}\cdot \frac{dW}{dr}=\frac{1}{8\pi \eta h}\cdot \frac{d}{dr}(2\pi r\gamma (r))=-\frac{1}{4\eta h}(\gamma (r)+r\frac{d\gamma }{dr})$$

Differential equation (26) for *dr*/*dt* = *V* = *const* resolved for *γ* yields:

27 $$\gamma (r)=4Vh\eta +\frac{C}{r},$$

where *C* is the integration constant. The dependence is similar to the predictions of the continuum theory (Figs 4–6): it accounts for growth of the line tension of the hydrophilic pore as the pore radius decreases. However, it fails to predict the local minimum of line tension at the radius of a few nanometers and an abrupt decrease of the line tension after transition into hydrophobic defect. Besides, the *r*(*t*) dependencies obtained from our MD modeling are not linear (Fig. 2B). More specifically, two straight lines with different slopes intersecting at *r* ∼ 1.3 nm, *t* ∼ 17 ns can provide a fair approximation of the time course for DOPC (Fig. 2B, red curve). The *r*(*t*) dependence for POPC can be roughly approximated by three straight lines with different slopes: i) from *r* ∼ 3 nm to *r* ∼ 1.4 nm (from *t* = 0 to *t* ∼ 18 ns); ii) a horizontal plateau at *r* ∼ 1.4 nm for about 5 ns (from *t* ∼ 18 ns to *t* ∼ 23 ns); iii) from *r* ∼ 1.4 nm to *r* = 0 (from *t* ∼ 23 ns to *t* ∼ 30 ns). The *r*(*t*) dependence for DMPC cannot be meaningfully decomposed into quasy-linear segments at all. The pore slowly reached the radius of *r* ∼ 1.9 nm corresponding to the local minimum of the pore energy (metastable state) (Fig. 4A, green curve), and then fluctuated around it until the end of the MD trajectory, remaining in the hydrophilic domain.

Molecular dynamic simulations with the use of potential of mean force (PMF) were used in ref.^17^ to analyze the pore energy landscape. PMF calculations are very sensitive to proper choice of a reaction coordinate. The authors used three different coordinates: pore radius, distance from the phosphate group of a certain lipid molecule to equatorial plane of the membrane, and water density in the pore lumen. Pores could be formed along any of these coordinates, but strong hysteresis existed between the pore opening and closure simulations. These results are in good agreement with our findings. Analysis of the trajectory of pore formation from an intact bilayer through a hydrophobic defect (Fig. 3A) reveals that the state of the system cannot be fully defined by a single coordinate: at the pore radius of *r* ∼ 0.7 nm, substantially different states characterized by hydrophobic belt heights of 2*L* = 0 and 2*L* = 2.5 nm have the same energy, i.e. are equally probable. Thus, pore radius alone does not define the system state, and this uncertainty manifests itself in the most interesting part of the trajectory — the region of transition between the hydrophobic defect and hydrophilic pore. Likewise, the half-height of the hydrophobic belt, which can be defined as a distance from the phosphate group of a certain lipid molecule to equatorial plane, cannot be used as a single coordinate fully describing the state of the system. Indeed, the hydrophilic pore with *L* = 0 can have different radii. Water density in the pore lumen is, in a sense, a parameter combining the radius and half-height of the hydrophobic belt. However, according to Marcelja approach, which we used for calculating the hydrophobic defect energy^28,29^, when the half-height of the hydrophobic belt is fixed, the order parameter of water, which is related to water density, monotonously changes with the belt radius. Thus, the radius and the half-height of the hydrophobic belt can be simultaneously changed in such a manner that the average water density in the pore lumen would remain constant, once again providing an example of two substantially different states of the system corresponding to the same value of the coordinate.

The hysteresis between pore opening and closure observed in the work ref.^17^ appears to reflect the existence of a metastable state. In our model, a hydrophilic pore having the radius of *r* ∼ 1–2 nm for DOPC, POPC, and DMPC (Fig. 4A) should correspond to this metastable state. In the above mentioned molecular dynamics studies of transversal pore formation^57,58^, the authors report absence of local minimum of pore free energy at small radii. However, the existence of metastable conducting defects of small radii is consistent with experimental data^45,46,56^. Besides, the physical reason behind the free energy local minimum at the pore radius of about 1–2 nm is quite straightforward: at this radius positive meridional and negative equatorial curvatures of the edge optimally compensate each other, thus optimizing the major (splay) contribution to the elastic energy (Table 2).

**Figure 7:**
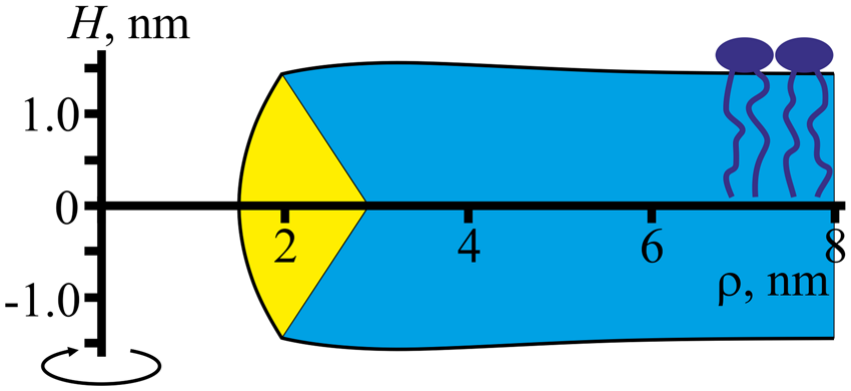
The shape of DOPC membrane in the vicinity of the pore edge (*r* = 1.5 nm) calculated in the framework of the continuum theory. Vertical monolayer region is shown in yellow; horizontal bilayer region is shown in blue. Elastic deformations extend to about 6 nm around the pore boundary.

The strong dependence of the results on the simulation system dimensions under imposed periodical boundary conditions observed in the ref.^17^ is consistent with the pore boundary shape we have calculated. Deformations around the pore boundary extend to the distance of ∼6 nm (Fig. 7), i.e. two pores do not interact as long as the distance between their edges exceeds ∼12 nm, which defines the required size of the simulation box.

The calculations in the ref.^17^ were performed for ≤ 512 lipid molecules, wherefrom the size of the box can be estimated to be about 13 × 13 nm. Thus, for a smaller system (64, 128, or 256 lipid molecules^17^) the influence of periodic boundary conditions has to be considerable indeed.

## Conclusions

We have developed a theoretical model of the pore edge structure. The model predicts a non-monotonous dependence of the line tension on the radius of the pore. Thus, for the same lipid composition, different values of the line tension can be determined experimentally, depending on the pore radius, for which it is determined. The calculated values of the line tension for the three lipids we investigated are close to those measured experimentally both for large pores and pores of the radii of the order of a few nanometers. The obtained result that the line tension depends on pore radius forces generalize the Derjaguin equation, Eq. (1), as:

28 $$E(r)=2\pi r\gamma (r)-\pi {r}^{2}{\sigma }_{0}$$

In the accompanying paper^19^ we further demonstrate that the line tension also depends on the lateral tension, i.e. *γ* = *γ*(*r*, σ_0_).

From the molecular dynamics simulations, it follows that the spontaneous closure of the pore passes through a hydrophobic defect. We assumed that the formation of the pore (a reverse process) also goes through the hydrophobic defect. This allowed developing a detailed theoretical model of the pore formation process. To the best of our knowledge, this is the first model to provide a continuous trajectory from an intact bilayer to a hydrophilic pore through hydrophobic defect, with gradual change of lipid orientation from vertical in the intact bilayer to horizontal in the hydrophilic pore.

It is shown that the transition of the hydrophobic defect to the hydrophilic pore is stochastic process, since in the vicinity of the transition point these two states have the same energy and they are separated by only a small energy barrier (units of *k*_*B*_ *T*). Because the luminal area of hydrophilic pore and the hydrophobic defect differs greatly, the transitions between these two states must be accompanied by a high-frequency noise of the electrical conductivity of the membrane, and the noise amplitude should be relatively stable. This noise referred to as a flicker is actually observed experimentally.

In the future, for general theoretical description of the process of transverse pore formation, the multi-component membrane case has to be analyzed^10,61^, and the dependence of the parameters on the method of formation of model membranes, including the organic solvent used in the process^10^, are to be taken into account. We plan to extend the developed model to address these cases.
